# Teaching Trans-Centric Curricular Content Using Modified Jigsaw

**DOI:** 10.15766/mep_2374-8265.11257

**Published:** 2022-05-24

**Authors:** Cynthia Zheng, Zoee D'Costa, Rob J. Zachow, Robert Lebeau, Gloria A. Bachmann

**Affiliations:** 1 Fourth-Year Medical Student, Rutgers Robert Wood Johnson Medical School; 2 Second-Year Medical Student, Rutgers Robert Wood Johnson Medical School; 3 Professor, Department of Biochemistry and Molecular Biology, Rutgers Robert Wood Johnson Medical School; 4 Director, Office for Advancing Learning, Teaching, and Assessment, and Director, Cognitive Skills Program, Rutgers Robert Wood Johnson Medical School; 5 Professor, Department of Obstetrics, Gynecology, and Reproductive Sciences; Director, Women's Health Institute; and Medical Director, PROUD Gender Center of New Jersey, Rutgers Robert Wood Johnson Medical School

**Keywords:** Jigsaw, Transgender, Discussion, Small Group (≤12), Endocrinology, Gender Identity, LGBTQ+, Physiology, Diversity, Equity, Inclusion

## Abstract

**Introduction:**

Transgender (trans) individuals have unique medical needs and difficulty accessing quality health care, exacerbated by inadequate provider knowledge. Incorporation of trans health care into medical school curricula has increased recently to address this gap. Jigsaw activities emphasize positive interdependence through structured cooperative learning, resulting in increased interest and self-confidence. We implemented a voluntary 2-hour modified jigsaw exercise on trans health care with changes designed to optimize the structure for medical students.

**Methods:**

The session was implemented both in person and virtually over 2 years with preclerkship medical students at the end of their endocrine/reproduction physiology course. The session featured a knowledge test with answer discussion followed by a clinical correlation—either a case discussion or video discussion. A pre- and posttest design compared students’ knowledge, attitudes, and beliefs.

**Results:**

Eighty-nine students participated. Their initial attitudes and beliefs regarding trans health care were highly positive and remained elevated. Participants showed increases in knowledge and self-confidence discussing gender identity and clinical care postsession. All expressed interest in further training and felt the session enhanced their understanding of trans health and reproductive physiology. On 1-year follow-up, students showed decreased knowledge and self-confidence in discussing trans health; however, scores remained higher than presession. Student surveys suggested formal integration of more trans health education into the curriculum.

**Discussion:**

Medical students increased their knowledge and self-confidence regarding trans medicine and felt the modified jigsaw exercise was an effective teaching method. The results suggest that ongoing education is an important tool in optimizing trans health care.

## Educational Objectives

By the end of this activity, learners will be able to:

1. Describe gender-affirming hormone regimens and expected physiologic effects.
2. Explain side effects of exogenous hormones and monitoring recommendations.
3. Identify key questions to ask when interviewing transgender patients.

## Introduction

Based on 2019 data, up to 2.7% of Americans identify as transgender (trans) or gender nonbinary.^1^ One challenge for trans and nonbinary folx is their experiences of erasure in health care due to the culmination of systemic oppression, implicit bias, and providers’ negative responses.^2–4^ Erasure can also mean lack of knowledge or perceived importance by providers, as illustrated by trans patients having to educate providers on their needs or tolerate invasive and insensitive questions.^2–5^ Providers report challenges in learning about resources and best practices for trans patients, specifically citing limited education in medical school or residency.^4^

Of the 131 U.S. medical schools included in the Association of American Medical Colleges’ Curriculum Inventory in 2018, 65% reported offering some kind of trans-related education.^6^ However, trans health care is often briefly covered, and students across all school years report that it is the least understood among LGBTQ+ content.^7,8^ During preclerkship years, schools indicate offering lectures,^9–12^ standardized patient encounters,^13^ film discussions,^14^ panel discussions, or combinations of methods.^7,15–18^ Some designs include discussion of trans medicine within physiology courses.^12,16,17^ Relevant *MedEdPORTAL* publications^14,16–18^ provide robust and multimodal activities including varied use of small-group learning techniques and case discussions. None, however, report using peer-learning approaches, such as the jigsaw method, that require active participation from every student.

The jigsaw method is a highly structured cooperative learning strategy that reorganizes the roles of students as learners and peer teachers to emphasize dependence on each other for success. In a traditional jigsaw model, students are assigned to groups of four to five. Content is divided into subtopics, and one individual from each group serves as an expert on a topic. Students independently research the area and discuss answers with other students who have been assigned the same content. Finally, students return to their original group to teach their respective topics.^19^ Sharing these roles and responsibilities, also known as positive interdependence,^20^ is fundamental to the positive effects of jigsaw methods, such as increased self-confidence, cooperation skills, and satisfaction coupled with decreased competitiveness, anxiety, and disinterest.^21^ Medical education has increasingly shifted to a learner-centered paradigm, and jigsaw methods have successfully been used in medical schools and graduate medical education to teach various topics, especially for tasks that can be completed in one session, to complement other active learning methods such as team-based learning.^22–24^

To optimize traditional jigsaw activities for medical students, modifications can be made to counter commonly cited obstacles such as required presession work^23^ and to promote higher-level reasoning beyond fact dissemination.^25^ Such an optional modified jigsaw session was designed that focused on gender-affirming hormone therapy during the endocrine/reproduction part of the first-year preclerkship curriculum. Furthermore, the session's focus on first-year students was intended to address a gap in the curriculum and provide a foundation for revisiting this material in the second-year curriculum.

## Methods

### Target Audience

We offered this session in 2020 and 2021 to all first-year students who were taking the M1 Endocrine and Reproduction Systems course to complement physiology concepts. We scheduled it toward the end of the course when students had broad foundations. In 2021, we opened the session to second-year students who did not participate in 2020. Since all accredited medical schools in the U.S. have education on endocrine and reproductive content, this session was designed to be easily incorporated into existing curricula.

### Presession Student Preparation

Prior to the session, all relevant endocrine and reproductive physiology lectures had been given by faculty. Topics included male and female reproductive anatomy and physiology, including the menstrual cycle, pregnancy, and adrenal physiology. Because students were given the option to watch lectures live or stream them asynchronously, not all students had completed every lecture prior to attending the session. With regard to clinical skills and patient interviewing, students had learned the basic structure of a full history and physical with limited application in a true clinical setting (e.g., interviewing patients in a clinic). Appendix A contains a suggested list of topics that students be familiar with prior to starting the session.

### Session Structure

**Figure 1.**
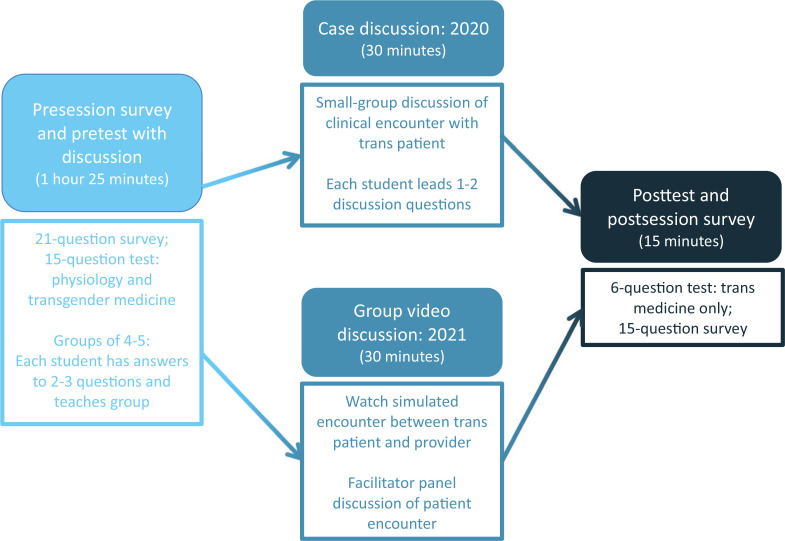
Session timeline.

The session totaled approximately 2 hours and was held in classrooms that allowed groups of five students to sit and discuss with one another without disturbing nearby groups. Due to the coronavirus pandemic, the 2021 session was held over Zoom, making use of breakout rooms, but overall structure remained the same compared to 2020 (Figure 1). Appendix A offers an outline of the activity. Appendix B includes the facilitator guide.

### Session Implementation

#### Pretest

We divided participants into groups of four to five. Participants individually completed a presession survey (Appendix C) followed by multiple-choice questions (Appendix D). We wrote all questions with five to six answer choices along with some associated clinical vignettes, consistent with the format of school examinations.

Endocrine and reproductive topics were chosen from course material and covered the hypothalamic-pituitary-gonadal axis, the menstrual cycle, steroidogenesis, reproductive embryology, hormone changes in pregnancy, and physiologic effects of hormones. Transgender-specific questions covered gender versus sexual identity, common feminizing and masculinizing hormone regimens, and expected effects of hormone regimens. We chose these topics based upon the work of Safer and Tangpricha^26^ and the University of San Francisco's transgender care guidelines^27^ because they served as a sufficient introduction to the topic and were connected to physiology. Questions were developed by the research team and independently reviewed and approved by the course director, who reviewed United States Medical Licensing Exam questions and multiple medical school exams, and the medical director of the PROUD Gender Center.

#### Test discussion

Once the pretest had been finished, each group of five students received student packets 1–5 (Appendices E–I), which had all pretest and discussion points divided amongst them, such that no single packet held all the answers. Using class notes and online resources, each student prepared and taught their assigned packet (one of the five) to group members. We allotted 1 hour and 25 minutes to complete this section. Participants then progressed to the patient case discussion in 2020 or to the video case discussion in 2021.

#### Clinical correlation

In 2020, each student received answers and notes to one or two of the discussion questions (Appendices E–I) and facilitated that portion of the discussion. This discussion described a clinical encounter with a transgender individual wishing to begin hormone therapy. Included in the case discussion were points on patient comfort and presenting and responding to information in a mindful manner. Thirty minutes were given for this case discussion. In 2021, all students watched a 12-minute simulated encounter between a trans patient and a care provider played by two faculty members (Appendix J). The simulated encounter detailed a trans male presenting for initial evaluation of abdominal pain, with discussion about gender identity, hormone therapy, social support, and substance use. Students then asked facilitators questions regarding the encounter and patient care, with some predetermined questions written by us to address similar learning objectives as 2020’s case discussion. The addition of the large-group video discussion was triggered by previous comments indicating a desire for more guidance regarding interviewing patients. The 2021 session was intended to have the case discussion follow the video session, but this was changed due to time restrictions.

#### Postsession

Students completed a multiple-choice posttest about hormone therapy (Appendices K and L) followed by a postsession survey (Appendix M).

### Faculty Preparation

Faculty facilitators from the departments of obstetrics and gynecology, endocrinology, and pediatric endocrinology and Robert Wood Johnson University Hospital's LGBT Health Navigators, all of whom were experienced in trans health care and part of the PROUD Gender Center of New Jersey, were given facilitator guides (Appendix B) and were present to answer questions. The LGBT Health Navigators identified as trans and additionally offered patient perspectives to the students.

Facilitators were sent the guide along with information detailing the session's structure and expectations at least 2 weeks prior to the session. All facilitators met 1 hour before the session for a briefing. During the briefing, we reviewed the facilitator guide, discussed possible student questions, and explained logistics. Faculty were given time to ask any questions, and all remaining time was used to independently review the guide. During the session, faculty facilitators and coordinators circulated.

### Session Evaluation

The presession survey assessed attitudes, beliefs, and prior experiences. The survey asked for prior experience interacting with trans individuals in personal and medical environments. We assessed degree of confidence discussing specific topics on a 100-point self-efficacy scale (0 = *cannot do at all,* 100 = *highly certain can do*) described by Bandura.^28^ We assessed attitudes and beliefs using a 5- or 7-point Likert scale (1 = *not at all comfortable* or *strongly disagree,* and 5 and 7 = *extremely comfortable* or *strongly agree,* respectively). Some questions were adapted from Sanchez and colleagues.^29^ We assessed knowledge via the pre- and posttests. The trans-specific subscore of the pretest was compared to the posttest score. Students were also able to provide written feedback.

We distributed a 1-year follow-up survey to the 2020 participants. The survey consisted of posttest, attitude, and comfort questions almost identical to those seen in the original session. Additional questions were added to gauge interest and behavior.

### Analysis

We used descriptive statistics to compare pre- and postsession scores using the Student two-sample *t* test. One-year follow-up data for 2020 were compared using one-way analysis of variance (ANOVA) and Tukey's post hoc test. We used StatPlus LE version 7.1.1.0 (AnalystSoft) and SPSS Statistics version 28.0.0.0 (IBM). We considered *p* < .05 to be statistically significant.

## Results

Eighty-nine of 340 students participated (26% participation rate; see the Table). Approximately half reported prior interactions with trans individuals, the majority of which occurred via school peers and/or friends. Only nine students had prior interactions in medical settings. Students also noted various societal obstacles they had personally witnessed, including stigma for gender expression, incorrect pronouns or names, difficulty accessing health care, and lack of overall institutional support.

**Table.**
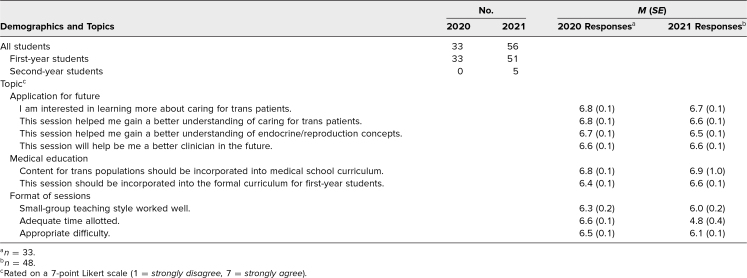
Demographics and Postsession Beliefs

**Figure 2.**
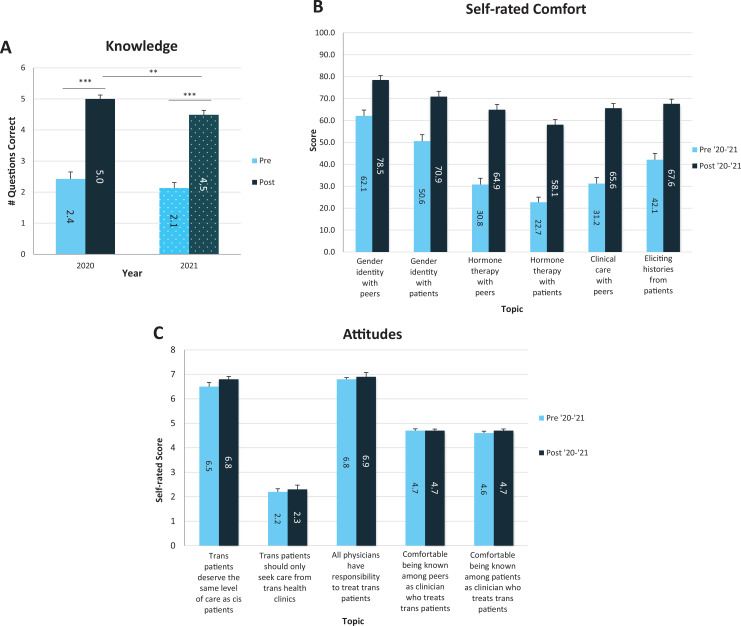
A: Pre- and posttest scores from 2020 (pre and post *n*s = 33) and 2021 (pre *n* = 53, post *n* = 47). ∗∗*p* = .01. ∗∗∗*p* < .001. B: Pre- and postsession self-rated comfort discussing various topics from 2020 (pre- and postsession *n*s = 33) and 2021 (presession *n* = 53, postsession *n* = 47). For all comparisons, *p* < .001. C: Pre- and postsession attitudes from 2020 (pre- and postsession *n*s = 33) and 2021 (presession *n* = 52, postsession *n* = 47). Questions regarding clinical care were based on a 7-point Likert scale (1 = *strongly disagree,* 7 = *strongly agree*). Questions regarding comfort were based on a 5-point Likert scale (1 = *not at all comfortable,* 5 = *extremely comfortable*). For all comparisons, *p* > .05. Error bars indicate standard error.

When presession and postsession knowledge, self-confidence, and attitudes were compared, knowledge of trans-specific topics (*p* < .001; Figure 2A) and self-confidence discussing trans health with peers and patients increased from pre- to postsession (*p* < .001 for all; Figure 2B). Similarly, confidence taking a patient history from a trans individual increased (*p* = .001; Figure 2B). Attitudes and beliefs were positive at both the beginning and end of the session (Figure 2C). We assessed participants’ attitudes towards trans-related content in medical education and their future careers (Table). Students reported that they wanted to learn more about trans medicine and that the session helped them gain better understanding of trans patient care and physiology. Similarly, students suggested the curriculum incorporate more trans-specific content, specifically including this session.

In comparing responses between 2020 and 2021, no difference was seen in attitudes. There were, however, differences in pre- and postsession comfort in taking histories and discussing gender-affirming hormone therapy with peers and patients, as well as in postsession comfort discussing gender identity with patients. In all instances, 2021 respondents rated themselves lower than 2020 respondents (*p* < .05 for all). Participants in 2021 also scored lower than those in 2020, averaging 4.5 out of 6.0 correct compared to 5.0 out of 6.0 correct on the posttest (*p* < .01).

Students provided feedback regarding overall impressions of the session. Students felt that the material was a suitable difficulty level. In 2021, students noted there was insufficient time, particularly for the group discussion. While most agreed that the small-group style worked, there was feedback regarding its effectiveness, specifically in feeling unprepared to teach the trans-specific topics. Some students reported reciting the explanations provided for those questions.

**Figure 3.**
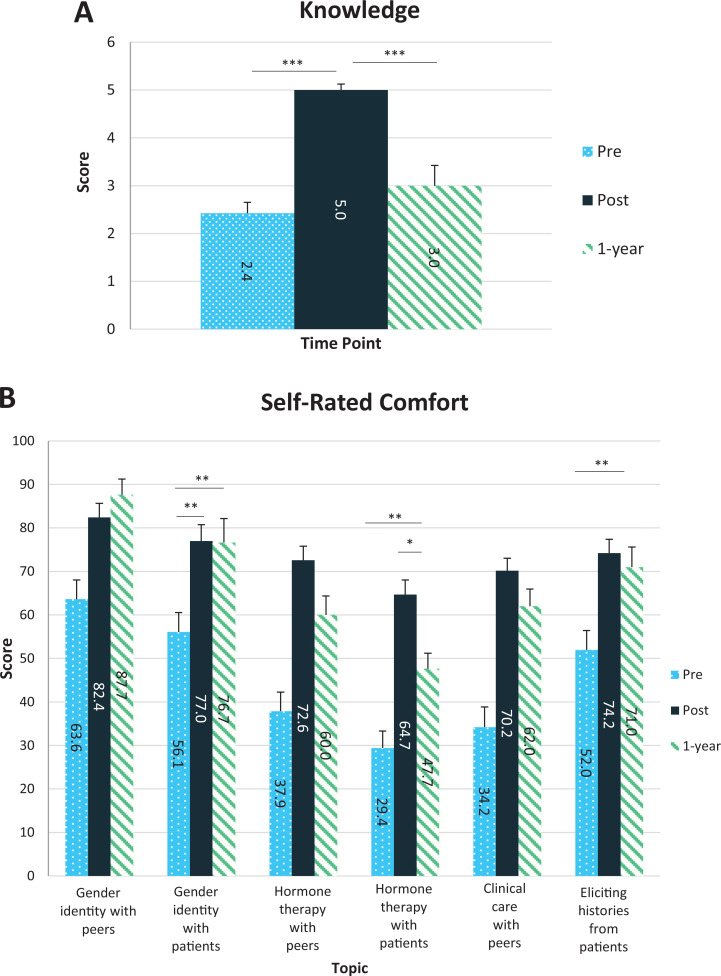
A: Pretest, posttest, and 1-year follow-up test scores (*n* = 33 for session, *n* = 11 for 1-year follow-up). ∗∗∗*p* < .001. B: Presession, postsession, and 1-year follow-up self-rated comfort discussing various topics (*n* = 33 for session, *n* = 15 for 1-year follow-up). ∗*p* = .05. ∗∗*p* = .01. All other comparisons are *p* > .001 or are insignificant unless stated otherwise. Error bars indicate standard error.

Twenty students from the 2020 cohort responded to the 1-year follow-up survey (46% response rate). Knowledge scores were lower than immediate posttest scores, 3.0 out of 6.0 correct compared to 5.0 out of 6.0 correct (Tukey's honestly significant difference [HSD]: *p* < .001) and slightly higher than pretest scores of 2.4 out of 6.0 (Figure 3A). Comfort discussing gender-affirming hormone therapy with patients decreased after 1 year but remained higher than pretest. ANOVA and Tukey's HSD did not otherwise show significant differences in comfort in comparing 1-year and postsession scores (Figure 3B). The majority agreed that the session helped for the final course exam and strongly agreed with the desire to learn more about trans health. Half of respondents reported that they independently learned more about trans health after the session.

## Discussion

To address a lack of longitudinal education on transgender health, we developed a modified jigsaw session on gender-affirming hormone therapy for preclerkship students to complement existing didactics on LGBTQ+ health. Changes were made to the traditional jigsaw format by exposing all students to every question and reducing the burden of outside research by providing the correct answers. These modifications were made to promote higher-order learning through analysis^25^ and to forge connections between course material and trans-centric content through highly structured cooperative learning. After attending the session, students demonstrated increased self-confidence and knowledge about basic clinical care regardless of prior experience with trans individuals. The positive effects on knowledge and self-confidence are consistent with effects seen with other didactics where cooperative learning is not the main mechanism of education.^7–12,16,29,30^ While we did not show an increase in positive attitudes, this was likely due to the highly positive attitudes seen in the presession survey, also reported by Najor and colleagues.^9^

In comparing the responses of 2020 and 2021, there were differences in posttest knowledge and comfort. The overall changes in comfort, however, remained comparable, indicating similar amounts of learning. Lower ratings in 2021 may have been due to discrepancies in session timing, with 2021 participants having more lectures remaining compared to 2020 participants, as well as to curricular modifications brought on by the pandemic. The differences may also have been due to the change in session clinical correlation. This change likely decreased overall engagement, as students were able to passively listen to facilitators. In the future, it would be optimal to extend the session by 30 minutes to allow time for both video and case discussions. To mitigate timing issues, facilitators should encourage transitions between sections of the activity. For example, independently answering questions should adhere to 1–1.5 minutes per question, as seen on licensing exams. A large-group case discussion could be done, as opposed to small groups individually, if the pretest discussion runs long. The results of posttest questions regarding cancer screening and procedures should be examined accordingly if that change is made. Despite these diminished effects, the online session still positively impacted students and offers a comparable experience to in-person implementation.

Regarding the format, students commented that more trans facilitators would enhance the experience. As the session had only two trans facilitators, their time was limited among groups. Other curricula have invited trans community members with great success.^16,17^ While not inherently necessary, having trans facilitators provided compelling patient perspectives. Both the video and the case discussion—particularly the video, in which a trans individual plays the role of the patient—convey aspects of the patient experience. Future iterations could have a combination of facilitators, so that each room has an experienced clinician and a trans community member, thus providing students with more resources for self-directed learning. In addition, most students found that teaching each other was effective. Some students did not feel prepared enough to present trans-specific content. The lack of presession requirements was deliberate in order to minimize time commitments and encourage participation. In the future, more time could be allotted for the answer preparation stage. If the session is a mandated activity, the implementation of a brief presession reading or video could reduce recitation of explanations.

On 1-year follow-up, knowledge and comfort regarding many trans medicine topics had decreased, indicating that a single session is insufficient to create long-term changes. Interestingly, comfort remained higher than pretest levels, suggesting a lasting effect on self-confidence and perceived capability. These findings combined with the students’ continued interest reflect both a desire and a need for longitudinal incorporation of trans-specific content into medical school curricula.^8–12,30^ In the future, trans medicine content can be further reinforced via an observed structured clinical examination, allowing students to apply the material.

A limitation of this session is that it was voluntary, so it is likely that only students who were already interested in the topic attended. Similarly, as the session occurred during a fast-paced course, even interested students may have been hesitant to participate. Due to the time constraints, especially in the 2021 session, students were not able to complete the small-group discussion, thus limiting potential retention. Further work should be done to directly assess attitudes and beliefs across a wider population. Although students endorsed independent learning after the session through self-reported surveys, it is unclear how long-term attitudes, perceptions, and behaviors in clinical settings would be impacted. Similarly, it is unclear precisely which aspects of the cooperative learning the students found enjoyable as the postsurvey session evaluated only whether they believed the peer teaching worked well. Future iterations should ask detailed and discerning questions regarding specific components, such as teamwork and communication skills, to better pinpoint effective areas. The change in both session modality (in person vs. virtual) and case discussion modality (small-group guided discussion vs. large-group video panel) did not allow for detailed comparison of differences. Questions were validated only by content experts and expert question-writers as we did not have the power to disseminate the questions to an adequate number of students, as is done for National Board of Medical Examiners tests. In addition, there was no control group—individuals who attended a lecture, for example—to compare the results to. Lastly, as the curriculum was piloted at a single institution with numerous faculty members who were highly experienced in trans health care, results may not be generalizable to additional institutions in various geographic locations.

This 2-hour modified jigsaw session encouraged students to actively discuss trans health care with one another. The cooperative learning was effective at disseminating knowledge and creating an enjoyable experience both in person and virtually. In addition, the format was effective at integrating physiology with clinical care. This student-driven session can serve as a starting point for curricular integration or as part of a series of didactics, complementing already-existing content. Starting trans education early in students’ careers will better enable them to become compassionate and competent providers for trans individuals.

## Appendices


Activity and Materials Outline.docxFacilitator Guide.docxPresession Survey.docxPretest Questions.docxStudent Packet 1.docxStudent Packet 2.docxStudent Packet 3.docxStudent Packet 4.docxStudent Packet 5.docxSimulated Transgender Patient Interview.mp4Posttest Questions.docxPosttest Answers.docxPostsession Survey.docx
*All appendices are peer reviewed as integral parts of the Original Publication.*
