# Supplementary material for: Teaching Trans-Centric Curricular Content Using Modified Jigsaw
Source: MedEdPORTAL. 2022 May 24;18:11257. doi: 10.15766/mep_2374-8265.11257 (PMC9127030; doi:10.15766/mep_2374-8265.11257)
Supplement: Supplementary file 1 — Activity and Materials Outline.docxFacilitator Guide.docxPresession Survey.docxPretest Questions.docxStudent Packet 1.docxStudent Packet 2.docxStudent Packet 3.docxStudent Packet 4.docxStudent Packet 5.docxSimulated Transgender Patient Interview.mp4Posttest Questions.docxPosttest Answers.docxPostsession Survey.docx [file mep_2374-8265.11257-s001.zip › I. Student Packet 5.docx]

**Pre-test Questions**

**Please note that you have answers to Q9, 14, and 15.**

You will be responsible for teaching your group the answers that you have in your own words. Explanations provided are meant to help guide your thought process. You are free to use outside resources (e.g., class lectures, research papers, society guidelines) to augment your teaching.

There are additional discussion questions at the end of some questions. Time-permitting, you should prepare answers to these questions as well and teach your group mates about these topics.

Use the following vignette for questions 1-4.

A 25-year-old masculine-appearing patient goes to the primary care physician to discuss starting hormone therapy. Assigned male at birth, she states that she has never identified being “male” even as a child and instead identifies as female. As a first step, she recently started going by a different name and using “she/her” as her pronouns. She finds her physical appearance particularly distressing and would like to feminize her features.

1. What is the most appropriate term to describe the gender identity of this patient?

A. Cisgender

B. Gay

C. Genderqueer

D. Transgender

E. Transvestite

After a thorough examination and discussion, the physician starts her on a feminizing hormone regimen.

2. What would be the best drugs to use in this scenario? (Answers listed as primary drug and adjuvant)

A. Finasteride and spironolactone

B. Oral estradiol and cortisol

C. Oral estradiol and progestins

D. Oral estradiol and spironolactone

E. Spironolactone and progestins

*Additional Discussion Question: what are the side effects of prolonged steroid use?*

3. What is a mechanism by which the adjuvant drug acts?

A. Central suppression of GnRH

B. Increasing release of prolactin

C. Inhibition of 5α-reductase

D. Androgen receptor antagonist

E. Stimulation of Sertoli cells to secrete more inhibin B

*Additional Discussion Question: what is the synthesis pathway of testosterone?*

4. Through the prescribed treatment regimen what physiological changes would you expect to occur?

A. Changes in bone shape

B. Changes in skin

C. Increased libido

D. Increased pitch of voice

E. Increased red blood cells

5. Which drug is incorrectly matched to its side effect?

A. Cyproterone – hyperprolactinemia

B. Estrogen – increased risk of venous thromboembolism

C. Finasteride - hypercalcemia

D. Spironolactone – hyperkalemia

E. Testosterone – polycythemia

*Additional Discussion Question: what tests could you do to monitor the side effects of these drugs? (e.g., how would you measure hyperkalemia?)*

6. _____ stimulates _______ which in turn secrete androgen binding protein and inhibin B.

A. FSH; Leydig cells

B. FSH; Sertoli cells

C. LH; Leydig cells

D. LH; Sertoli cells

E. GnRH; pituitary

*Additional Discussion Question: what do inhibin B and androgen binding protein do?*

7. A 16-year-old male-appearing patient goes to the endocrinologist due to an “abnormal” puberty. While he has normal testes and claims that his karyotype at birth showed 46, XY, he complains of sparse body hair and abnormal breast growth. Blood tests show an elevated testosterone:DHT ratio with normal testosterone levels. All other steroids were also at normal levels. Administration of what drug would induce a similar clinical presentation?

A. 5α-reductase inhibitor

B. Androgen receptor antagonist

C. CYP17 inhibitor

D. Glucocorticoid

E. GnRH antagonist

Use the following vignette for questions 8-10.

A 50-year-old trans male patient comes into the office for a routine visit to monitor his hormone levels. He has no past medical history of surgeries.

8. Prior to having started testosterone, which of the following hormone levels reflects changes in the late follicular phase compared to mid follicular phase? (↑,increased; ↓, decreased; N, no change) (E2=estrogen, FSH=follicle-stimulating hormone)

E2 FSH Inhibin B Activin

A. ↑ ↑ ↑ ↑

B. ↑ ↓ ↑ ↓

C. ↑ ↑ ↑ ↓

D. ↑ ↓ ↓ ↑

E. ↑ ↑ ↓ ↑

9. Once he began administering parenteral testosterone (the typical regimen for female-to-male therapy), it directly acts on various tissues. What physiological effects would be expected to occur within the first 6 months?

*A. Fat redistribution

B. Hoarseness of voice

C. Typical cis male pattern baldness

D. Typical cis male pattern facial hair and beard

E. Vaginal enlargement

**A is the correct answer**. Fat redistribution is one of the changes seen in approximately 1-6 months. Patients also report increased acne and deepening of voices. Height is unchanged for reasons similar to those in female-to-male hormone therapy.

B is incorrect as voice hoarseness does not occur though deepening will

C is incorrect as changes in hair typically take longer than 6 months to occur

D is incorrect for similar reasons as C

E is incorrect as the vagina does not change size. The clitoris, however, may increase in size

10. In addition to the physiological changes described in question 8, the patient reported the cessation of his menses. This effect is due to negative feedback of GnRH. What role does testosterone play in this mechanism?

A. Metabolism into androstenedione by CYP17 in granulosa cells

B. Metabolism into androstenedione by CYP17 in theca cells

C. Metabolism into estrogen by CYP19 in granulosa cells

D. Metabolism into estrogen by CYP19 in theca cells

E. Metabolism into pregnenolone by CYP11A in granulosa cells

F. Metabolism into pregnenolone by CYP11A in theca cells

*Additional Discussion Question: How do theca and granulosa cells produce their respective hormones?*

11. A 6-year-old boy is brought to his pediatrician due to the recent appearance of public and axillary hair. His mother reports a healthy pregnancy, and the child’s development and general health prior to the appearance of body hair was ‘normal’. Physical exam shows a well-developed phenotypical male child with descended testes. His vitals are all within normal ranges. Which of the following would best explain the most likely etiology of this boy’s symptoms?

A. Constitutively active FSH receptor

B. CYP17 loss-of function mutation

C. CYP19 gain-of-function mutation

D. Gonadotropin-secreting pituitary adenoma

E. LH receptor loss-of-function mutation

12. A 23-year-old woman presents to her gynecologist due to oligomenorrhea over the past year (periods occurring approximately every 35-40 days, with some missed periods), the appearance of facial acne, and facial hair over her upper lip. Her prior medical history is unremarkable, she has never been pregnant. Physical exam shows a well-developed female, BMI = 26 kg/m^2^. Vitals include BP of 140/65 mm Hg, pulse 70/min, temp 37 °C, SpO_2_ 99%. If measured and relative to normal, which of the following blood lab panels would most likely be obtained in this woman? (↑,increased; ↓, decreased; N, no change) (SHBG, sex hormone binding globulin; HDL, high density lipoprotein; FSH, follicle-stimulating hormone)

SHBG FSH Free testosterone HDL

A. ↑ ↑ ↑ ↑

B. ↑ ↓ ↓ ↑

C. ↓ ↓ ↑ ↓

D. ↓ ↑ ↑ ↓

E. N ↓ ↓ N

F. N ↑ ↓ N

*Additional Discussion Question: what are treatment options for polycystic ovary syndrome?*

13. During fetal development, either the Müllerian duct or the Wolffian duct will not regress and progress into gonads. In biological males, SRY and TDF stimulate the testis to secrete testosterone and AMH. What will happen to the ducts?

A. Müllerian duct becomes internal gonads, and Wolffian duct becomes external gonads.

B. Müllerian duct becomes internal gonads, and Wolffian duct regresses.

C. Wolffian duct and Müllerian duct become internal gonads.

D. Wolffian duct becomes internal gonads, and Müllerian duct becomes external gonads.

E. Wolffian duct becomes internal gonads, and Müllerian duct regresses.

*Additional Discussion Question: What do the Wolffian and Müllerian ducts become, respectively, in adults? What is the effect of DHT on genital development?*

14. Your patient, a trans man, and his partner are in the process of starting a family. Having temporarily stopped testosterone more than 6 months prior to his pregnancy, he is currently 25 weeks pregnant. Which of the following describes the effects of E_2_ and progesterone on the following systems?

Na+ reabsorption Water reabsorption Minute ventilation

A. ↑ ↑ ↓

B. ↑ ↓ ↓

*C. ↑ ↑ ↑

D. ↓ ↓ ↓

E. ↓ ↓ ↑

**C is the correct answer**. E_2_ stimulates the renin-angiotensin system, which stimulates increased sodium and water reabsorption. Progesterone and E_2_ also stimulate AVP, arginine vasopressin, which results in further water reabsorption. Progesterone stimulates medullary respiratory centers and carotid body chemoreceptors to increase ventilatory drive and minute ventilation. To note, there have been some studies done examining transgender men during pregnancy, delivery, and birth outcomes. No conclusive evidence has yet been found to indicate adverse outcomes correlated with prior testosterone use.

A is incorrect as minute ventilation and venilatory drive will increase due to progesterone stimulating medullary respiratory centers and carotid body chemoreceptors

B is incorrect as water reabsorption would increase due to E_2_ stimulating RAS, and progesterone and E2 stimulating AVP.

D is incorrect for similar reasons as A and B. Na+ reabsorption would also be increased due to RAS and AVP activation

E is incorrect for similar reasons as D.

*Additional Discussion Question: Why is increased Na+ and water reabsorption, increased minute ventilation, and vasodilation important during pregnancy?*

15. Your patient from question 14 is now 35 weeks pregnant, and you measure his hormone levels to check on his progress. Which of the following hormone profiles would you expect to see that will prepare the uterus for delivery? (P_4_ = progesterone, E_3_ = estriol, E_2_ = estradiol-17β)

A. E_2_ > E_3_

B. E_3_ > P_4_

*C. E_3_ > E_2_

D. P_4_ > E_2_

E. P_4_ > E_3_

**C is the correct answer**. Throughout the pregnancy, E_3_ and E_2_ levels remain relatively similar until the third trimester where there will be almost a 10-fold increase in E_3_ levels due to an increase in DHEA levels (mostly from the fetal adrenal glands). This activates the placental ER to begin expression of myometrial contractile proteins.

A is incorrect as E_3_ will drastically increase compared to E_2_

B is incorrect as P_4_ is present in higher levels than E_3_

D is incorrect because P_4_ does not play a role in preparing for contractions, instead exerting a quiescing effect within myometrium. It is correct that P_4_ is present in higher levels than both E_3_ and E_2_

E is incorrect for similar reasons as D

*Additional Discussion Question: What effect do oxytocin, prostaglandins, and relaxin play during labor and delivery?*

**Case Discussions**

**Please note that you only have the answers to the sixth and seventh questions provided below**

Use the discussion points to guide a discussion within your group. If you have outside resources from the previous section that are helpful during these discussions, please feel free to use and share them.

You knock and walk into the exam room to see your next patient, who is a new patient. Upon first impression, the patient appears to be male-presenting and generally well although fidgety and tense while sitting in the chair. How would you start the conversation? (Think PCM)

The patient states that her legal name is John Smith but has recently started going by Diane. She is 32 and uses she/her pronouns. When you ask what brings her to the clinic, she explains that she wants to start gender-affirming hormone therapy. What additional information would you want to know from the patient regarding hormone therapy?

Diane explains that she has researched some things on the internet and tried to go to her previous primary care physician to start hormone therapy, but they had no experience in the area. How would you explain hormone therapy to Diane? Be sure to include what physiologic effects would NOT be seen and adverse effects

After hearing your explanation, Diane still wants to start therapy. You move on to complete the past medical history and rest of the clinical interview. What specific information would you not want to miss in each of the sections?

Past Medical History:

Past Surgical History:

Family History:

Medications:

Allergies:

Other:

You then take a detailed Social History. What information would you want to know?

Social History:

Sexual history:

You finish taking a full history of Diane. You then explain the typical treatment regimen for a male-to-female transition. After getting the results for an initial health screen, you will start her on low doses of oral estradiol and oral spironolactone. She will have to come in every three months to measure blood hormone levels. The goal will be to lower testosterone levels to the female range. When she comes in for her checkups, what parameters should be measured?

*Estradiol, testosterone, potassium (spironolactone side effect), BP (spironolactone side effect), blood glucose, prolactin, triglycerides*

At the conclusion of her exam, she mentions that she would like some feminization surgeries in the future. You counsel her on various options including facial feminization surgeries, breast augmentation, and genital reconstruction surgeries like vaginoplasty and orchiectomy (removal of the testes). How do surgeries affect screening practices? Consider both transmen and transwomen.

*Screening for adult natal male (typically after age 50): bone density, prostate cancer, testicular cancer, colorectal cancer*

*Screening for adult natal female (typically after age 50): bone density, breast cancer, cerivical cancer, colorectal cancer*

*Key Points*

- *Check for the organs that a person has REGARDLESS of hormone therapy*
- *There are some recommendations that transwomen should get breast cancer screening due to the exposure of estrogen*
