# Supplementary material for: Teaching Trans-Centric Curricular Content Using Modified Jigsaw
Source: MedEdPORTAL. 2022 May 24;18:11257. doi: 10.15766/mep_2374-8265.11257 (PMC9127030; doi:10.15766/mep_2374-8265.11257)
Supplement: Supplementary file 1 — Activity and Materials Outline.docxFacilitator Guide.docxPresession Survey.docxPretest Questions.docxStudent Packet 1.docxStudent Packet 2.docxStudent Packet 3.docxStudent Packet 4.docxStudent Packet 5.docxSimulated Transgender Patient Interview.mp4Posttest Questions.docxPosttest Answers.docxPostsession Survey.docx [file mep_2374-8265.11257-s001.zip › L. Posttest Answers.docx]

1. A 34-year-old male-appearing patient comes to your clinic wanting to start gender affirming hormone therapy to feminize body and facial features. What regimen would you start him on?

A. Cyproterone with spironolactone

B. Finasteride with spironolactone

C. Oral estradiol alone

*D. Oral estradiol with cyproterone

E. Spironolactone alone

2. What must the physician strongly advise the patient in question 1 do?

A. Avoid fatty foods due to increased LDL

B. Finalize family building plans before starting regimen

C. Limit sugar intake due to increased HbA1c

*D. Smoking cessation due to the increased risk of venous thromboembolism

E. Watch for signs of polycythemia including headache, blurry vision, or flushed skin

3. After a year of being on this regimen, your patient comes back for a regular follow-up. What physiological changes would you expect to have occurred?

A. Changes in voice

B. Increased acne

C. Increased hair loss

D. Increased libido

*E. Redistribution of fat

4. A 25-year-old transgender male has been on parenteral testosterone for the past 8 months. During a regular screening, what parameter must be checked in addition to total testosterone at every 6-month visit?

A. Bone density

B. Estradiol

C. HbA1c

*D. Hemoglobin and hematocrit

E. Lipids

5. What physiologic effects would not be expected in the patient from question 4?

A. Clitoral enlargement

B. Deepening of the voice

C. Increased muscle mass

*D. Vaginal enlargement

E. Skin changes

6. You have a patient who is a 60-year-old trans female patient who is currently on low doses of estrogen and finasteride. Her past surgical history is significant for vaginoplasty, orchiectomy, and facial feminization surgeries. She has no family history of cancer. Which of the following screening tests would you recommend?

A. Liver cancer

B. Cervical cancer

C. Ovarian cancer

*D. Prostate cancer

E. Testicular cancer
