# Supplementary material for: Teaching Trans-Centric Curricular Content Using Modified Jigsaw
Source: MedEdPORTAL. 2022 May 24;18:11257. doi: 10.15766/mep_2374-8265.11257 (PMC9127030; doi:10.15766/mep_2374-8265.11257)
Supplement: Supplementary file 1 — Activity and Materials Outline.docxFacilitator Guide.docxPresession Survey.docxPretest Questions.docxStudent Packet 1.docxStudent Packet 2.docxStudent Packet 3.docxStudent Packet 4.docxStudent Packet 5.docxSimulated Transgender Patient Interview.mp4Posttest Questions.docxPosttest Answers.docxPostsession Survey.docx [file mep_2374-8265.11257-s001.zip › E. Student Packet 1.docx]

**Pre-test Questions**

**Please note that you have answers to Q1, 8, and 12.**

You will be responsible for teaching your group the answers that you have in your own words. Explanations provided are meant to help guide your thought process. You are free to use outside resources (e.g., class lectures, research papers, society guidelines) to augment your teaching.

There are additional discussion questions at the end of some questions. Time-permitting, you should prepare answers to these questions as well and teach your group mates about these topics.

Use the following vignette for questions 1-4.

A 25-year-old masculine-appearing patient goes to the primary care physician to discuss starting hormone therapy. Assigned male at birth, she states that she has never identified being “male” even as a child and instead identifies as female. As a first step, she recently started going by a different name and using “she/her” as her pronouns. She finds her physical appearance particularly distressing and would like to feminize her features.

1. What is the most appropriate term to describe the gender identity of this patient?

A. Cisgender

B. Gay

C. Genderqueer

*D. Transgender

E. Transvestite

**D is correct**. Transgender is currently the most accepted form of describing an individual whose gender identity is different from the sex assigned at birth.

A is incorrect as cisgender refers to individuals who identify with their assigned sex.

B is incorrect as “gay” describes a sexual orientation, which refers to whom the individual is attracted to. Gender identity refers to an individual’s internal sense of where they are on the gender spectrum.

C is incorrect as it typically refers to individuals who feel that are both male and female or neither. Since the patient currently identifies as female, it would not be the best option. However of note, the best way to determine how your patient identifies is to directly ask.

E is incorrect as “transvestite” is largely outdated and considered offensive by many in the LGBTQ+ community. Cross-dresser has replaced the term to typically refer to a male who occasionally wears clothes, makeup, accessories that are associated with females as a form of gender identity.

After a thorough examination and discussion, the physician starts her on a feminizing hormone regimen.

2. What would be the best drugs to use in this scenario? (Answers listed as primary drug and adjuvant)

A. Finasteride and spironolactone

B. Oral estradiol and cortisol

C. Oral estradiol and progestins

D. Oral estradiol and spironolactone

E. Spironolactone and progestins

*Additional Discussion Question: what are the side effects of prolonged steroid use?*

3. What is a mechanism by which the adjuvant drug acts?

A. Central suppression of GnRH

B. Increasing release of prolactin

C. Inhibition of 5α-reductase

D. Androgen receptor antagonist

E. Stimulation of Sertoli cells to secrete more inhibin B

*Additional Discussion Question: what is the synthesis pathway of testosterone?*

4. Through the prescribed treatment regimen what physiological changes would you expect to occur?

A. Changes in bone shape

B. Changes in skin

C. Increased libido

D. Increased pitch of voice

E. Increased red blood cells

5. Which drug is incorrectly matched to its side effect?

A. Cyproterone – hyperprolactinemia

B. Estrogen – increased risk of venous thromboembolism

C. Finasteride - hypercalcemia

D. Spironolactone – hyperkalemia

E. Testosterone – polycythemia

*Additional Discussion Question: what tests could you do to monitor the side effects of these drugs? (e.g., how would you measure hyperkalemia?)*

6. _____ stimulates _______ which in turn secrete androgen binding protein and inhibin B.

A. FSH; Leydig cells

B. FSH; Sertoli cells

C. LH; Leydig cells

D. LH; Sertoli cells

E. GnRH; pituitary

*Additional Discussion Question: what do inhibin B and androgen binding protein do?*

7. A 16-year-old male-appearing patient goes to the endocrinologist due to an “abnormal” puberty. While he has normal testes and claims that his karyotype at birth showed 46, XY, he complains of sparse body hair and abnormal breast growth. Blood tests show an elevated testosterone:DHT ratio with normal testosterone levels. All other steroids were also at normal levels. Administration of what drug would induce a similar clinical presentation?

A. 5α-reductase inhibitor

B. Androgen receptor antagonist

C. CYP17 inhibitor

D. Glucocorticoid

E. GnRH antagonist

Use the following vignette for questions 8-10.

A 50-year-old trans male patient comes into the office for a routine visit to monitor his hormone levels. He has no past medical history of surgeries.

8. Prior to having started testosterone, which of the following hormone levels reflects changes in the late follicular phase compared to mid follicular phase? (↑,increased; ↓, decreased; N, no change) (E2=estrogen, FSH=follicle-stimulating hormone)

E2 FSH Inhibin B Activin

A. ↑ ↑ ↑ ↑

B. ↑ ↓ ↑ ↓

C. ↑ ↑ ↑ ↓

D. ↑ ↓ ↓ ↑

*E. ↑ ↑ ↓ ↑

**E is the correct answer**. Early in the follicular phase, E2 exerts negative feedback on the GnRH pulse generator, and preovulatory granulosa cells produce inhibin B which inhibits activin. Towards the latter half of the follicular phase, E2 levels increase to a point that it now exerts positive feedback on FSH secretion. Inhibin B levels also drop and allow activin to further stimulate FSH secretion.

A is incorrect as inhibin B levels would drop

B is incorrect as FSH increases due to positive feedback from E2, and inhibin B decreases

C is incorrect as activin increases due to decreased inhibin B

D is incorrect as FSH increases

9. Once he began administering parenteral testosterone (the typical regimen for female-to-male therapy), it directly acts on various tissues. What physiological effects would be expected to occur within the first 6 months?

A. Fat redistribution

B. Hoarseness of voice

C. Typical cis male pattern baldness

D. Typical cis male pattern facial hair and beard

E. Vaginal enlargement

10. In addition to the physiological changes described in question 8, the patient reported the cessation of his menses. This effect is due to negative feedback of GnRH. What role does testosterone play in this mechanism?

A. Metabolism into androstenedione by CYP17 in granulosa cells

B. Metabolism into androstenedione by CYP17 in theca cells

C. Metabolism into estrogen by CYP19 in granulosa cells

D. Metabolism into estrogen by CYP19 in theca cells

E. Metabolism into pregnenolone by CYP11A in granulosa cells

F. Metabolism into pregnenolone by CYP11A in theca cells

*Additional Discussion Question: How do theca and granulosa cells produce their respective hormones?*

11. A 6-year-old boy is brought to his pediatrician due to the recent appearance of public and axillary hair. His mother reports a healthy pregnancy, and the child’s development and general health prior to the appearance of body hair was ‘normal’. Physical exam shows a well-developed phenotypical male child with descended testes. His vitals are all within normal ranges. Which of the following would best explain the most likely etiology of this boy’s symptoms?

A. Constitutively active FSH receptor

B. CYP17 loss-of function mutation

C. CYP19 gain-of-function mutation

D. Gonadotropin-secreting pituitary adenoma

E. LH receptor loss-of-function mutation

12. A 23-year-old woman presents to her gynecologist due to oligomenorrhea over the past year (periods occurring approximately every 35-40 days, with some missed periods), the appearance of facial acne, and facial hair over her upper lip. Her prior medical history is unremarkable, she has never been pregnant. Physical exam shows a well-developed female, BMI = 26 kg/m^2^. Vitals include BP of 140/65 mm Hg, pulse 70/min, temp 37 °C, SpO_2_ 99%. If measured and relative to normal, which of the following blood lab panels would most likely be obtained in this woman? (↑,increased; ↓, decreased; N, no change) (SHBG, sex hormone binding globulin; HDL, high density lipoprotein; FSH, follicle-stimulating hormone)

SHBG FSH Free testosterone HDL

A. ↑ ↑ ↑ ↑

B. ↑ ↓ ↓ ↑

*C. ↓ ↓ ↑ ↓

D. ↓ ↑ ↑ ↓

E. N ↓ ↓ N

F. N ↑ ↓ N

**C is correct.** Her symptoms of recent onset acne and hirsutism with oligomenorrhea suggest androgen excess. This makes choices B, E, F less likely. Elevated testosterone decreases the production of the androgen binding protein SHBG, enabling higher levels of serum free testosterone. Testosterone exerts negative feedback on FSH (and LH) secretion, which is disrupting the normal neuroendocrine control of ovarian function, thus impacting her menstrual cycle. Finally, elevated testosterone has the potential to lower HDL levels. Based upon her BMI, she appears to be overweight; more testing should investigate the possibility that this woman has developed polycystic ovary syndrome.

A is incorrect as SHGB production would be inhibited by testosterone. FSH would also decrease, and HDL would be lowered.

B is incorrect as SHBG production is inhibited, and free testosterone would be increased

D is incorrect as FSH is inhibited through negative feedback

E is incorrect as SHBG and HDL are affected and free testosterone would increase.

F is incorrect as SHBG and HDL are affected, free testosterone would increase, and FSH would decrease.

*Additional Discussion Question: what are treatment options for polycystic ovary syndrome?*

13. During fetal development, either the Müllerian duct or the Wolffian duct will not regress and progress into gonads. In biological males, SRY and TDF stimulate the testis to secrete testosterone and AMH. What will happen to the ducts?

A. Müllerian duct becomes internal gonads, and Wolffian duct becomes external gonads.

B. Müllerian duct becomes internal gonads, and Wolffian duct regresses.

C. Wolffian duct and Müllerian duct become internal gonads.

D. Wolffian duct becomes internal gonads, and Müllerian duct becomes external gonads.

E. Wolffian duct becomes internal gonads, and Müllerian duct regresses.

*Additional Discussion Question: What do the Wolffian and Müllerian ducts become, respectively, in adults? What is the effect of DHT on genital development?*

14. Your patient, a trans man, and his partner are in the process of starting a family. Having temporarily stopped testosterone more than 6 months prior to his pregnancy, he is currently 25 weeks pregnant. Which of the following describes the effects of E_2_ and progesterone on the following systems?

Na+ reabsorption Water reabsorption Minute ventilation

A. ↑ ↑ ↓

B. ↑ ↑ ↓

C. ↑ ↑ ↑

D. ↓ ↓ ↓

E. ↓ ↓ ↑

*Additional Discussion Question: Why is increased Na+ and water reabsorption, increased minute ventilation, and vasodilation important during pregnancy?*

15. Your patient from question 14 is now 35 weeks pregnant, and you measure his hormone levels to check on his progress. Which of the following hormone profiles would you expect to see that will prepare the uterus for delivery? (P_4_ = progesterone, E_3_ = estriol, E_2_ = estradiol-17β)

A. E_2_ > E_3_

B. E_3_ > P_4_

C. E_3_ > E_2_

D. P_4_ > E_2_

E. P_4_ > E_3_

*Additional Discussion Question: What effect do oxytocin, prostaglandins, and relaxin play during labor and delivery?*

**Case Discussion**

**Please note that you only have the answers to the first 2 questions provided below**

Use the discussion points to guide a discussion within your group. If you have outside resources from the previous section that are helpful during these discussions, please feel free to use and share them.

You knock and walk into the exam room to see your next patient, who is a new patient. Upon first impression, the patient appears to be masculine-presenting and generally well although fidgety and tense while sitting in the chair. How would you start the conversation?

*Hi, my name is XYZ and I’m a first-year medical student ABC Medical School. Can I have you confirm your name and age for me? And how would you like to be addressed? My pronouns are XYZ; what are your pronouns?*

*Key points:*

- *Confirmation of legal name*
- *Name that patient goes by and make note of that on patient chart*
- *Pronouns: avoid saying “preferred” as it isn’t merely a preference*
- *If you’re ever unsure about something, ask (for example, although this patient is masculine-presenting, you should not assume the patient uses he/him pronouns)*

The patient states that her legal name is John Smith but has recently started going by Diane. She is 32 and uses she/her pronouns. When you ask what brings her to the clinic, she explains that she wants to start gender-affirming hormone therapy. What additional information would you want to know from the patient regarding hormone therapy?

- *Patient’s understanding of what hormone therapy is: effects, risks, benefits*
- *Patient expectations of effects (magnitude of effects, how fast effects are expected to be seen): for example, if the patient wants to start masculinizing hormone therapy, do they expect to immediately begin growing facial hair or grow an Adam’s apple?*
- *Patient’s goals for hormone therapy: what kind of physical effects do they want to see?*
- *How long they have considered hormone therapy*
- *Prior discussions about hormone therapy: have they spoke about it friends, other members of the trans community, other providers, etc.?*
- *Prior experience with hormones: previous provider, personal purchase of hormones, etc.*

Diane explains that she has researched some things on the internet and tried to go to her previous primary care physician to start hormone therapy, but they had no experience in the area. How would you explain hormone therapy to Diane? Be sure to include what physiologic effects would NOT be seen and adverse effects

After hearing your explanation, Diane still wants to start therapy. You move on to complete the past medical history and rest of the clinical interview. What specific information would you not want to miss in each of the sections?

Past Medical History:

Past Surgical History:

Family History:

Medications:

Allergies:

Other:

You then take a detailed Social History. What information would you want to know?

Social History:

Sexual history:

You finish taking a full history of Diane. You then explain the typical treatment regimen for a male-to-female transition. After getting the results for an initial health screen, you will start her on low doses of oral estradiol and oral spironolactone. She will have to come in every three months to measure blood hormone levels. The goal will be to lower testosterone levels to the female range. When she comes in for her checkups, what parameters should be measured?

At the conclusion of her exam, she mentions that she would like some feminization surgeries in the future. You counsel her on various options including facial feminization surgeries, breast augmentation, and genital reconstruction surgeries like vaginoplasty and orchiectomy (removal of the testes). How do surgeries affect screening practices? Consider both transmen and transwomen.
