# Supplementary material for: Teaching Trans-Centric Curricular Content Using Modified Jigsaw
Source: MedEdPORTAL. 2022 May 24;18:11257. doi: 10.15766/mep_2374-8265.11257 (PMC9127030; doi:10.15766/mep_2374-8265.11257)
Supplement: Supplementary file 1 — Activity and Materials Outline.docxFacilitator Guide.docxPresession Survey.docxPretest Questions.docxStudent Packet 1.docxStudent Packet 2.docxStudent Packet 3.docxStudent Packet 4.docxStudent Packet 5.docxSimulated Transgender Patient Interview.mp4Posttest Questions.docxPosttest Answers.docxPostsession Survey.docx [file mep_2374-8265.11257-s001.zip › G. Student Packet 3.docx]

**Pre-test Questions**

**Please note that you have answers to Q4, 10, and 11.**

You will be responsible for teaching your group the answers that you have in your own words. Explanations provided are meant to help guide your thought process. You are free to use outside resources (e.g., class lectures, research papers, society guidelines) to augment your teaching.

There are additional discussion questions at the end of some questions. Time-permitting, you should prepare answers to these questions as well and teach your group mates about these topics.

Use the following vignette for questions 1-4.

A 25-year-old masculine-appearing patient goes to the primary care physician to discuss starting hormone therapy. Assigned male at birth, she states that she has never identified being “male” even as a child and instead identifies as female. As a first step, she recently started going by a different name and using “she/her” as her pronouns. She finds her physical appearance particularly distressing and would like to feminize her features.

1. What is the most appropriate term to describe the gender identity of this patient?

A. Cisgender

B. Gay

C. Genderqueer

D. Transgender

E. Transvestite

After a thorough examination and discussion, the physician starts her on a feminizing hormone regimen.

2. What would be the best drugs to use in this scenario? (Answers listed as primary drug and adjuvant)

A. Finasteride and spironolactone

B. Oral estradiol and cortisol

C. Oral estradiol and progestins

D. Oral estradiol and spironolactone

E. Spironolactone and progestins

*Additional Discussion Question: what are the side effects of prolonged steroid use?*

3. What is a mechanism by which the adjuvant drug acts?

A. Central suppression of GnRH

B. Increasing release of prolactin

C. Inhibition of 5α-reductase

D. Androgen receptor antagonist

E. Stimulation of Sertoli cells to secrete more inhibin B

*Additional Discussion Question: what is the synthesis pathway of testosterone?*

4. Through the prescribed treatment regimen what physiological changes would you expect to occur?

A. Changes in bone shape

*B. Changes in skin

C. Increased libido

D. Increased pitch of voice

E. Increased red blood cells

**B is correct**. Estrogen will induce effects including decreased oiliness and softening of skin, breath tissue growth, redistribution of fat, and decreased facial and body hair.

A is incorrect as estrogen will not reverse the prior effects of androgens on bone structure.

C is incorrect. Patients will in fact report reduce libido.

D is incorrect for a reason similar to B. Estrogen has no effect on already formed cartilage structures. As this patient is an adult, bones will not be actively growing.

E is incorrect. That is an important side effect of testosterone treatment.

5. Which drug is incorrectly matched to its side effect?

A. Cyproterone – hyperprolactinemia

B. Estrogen – increased risk of venous thromboembolism

C. Finasteride - hypercalcemia

D. Spironolactone – hyperkalemia

E. Testosterone – polycythemia

*Additional Discussion Question: what tests could you do to monitor the side effects of these drugs? (e.g., how would you measure hyperkalemia?)*

6. _____ stimulates _______ which in turn secrete androgen binding protein and inhibin B.

A. FSH; Leydig cells

B. FSH; Sertoli cells

C. LH; Leydig cells

D. LH; Sertoli cells

E. GnRH; pituitary

*Additional Discussion Question: what do inhibin B and androgen binding protein do?*

7. A 16-year-old male-appearing patient goes to the endocrinologist due to an “abnormal” puberty. While he has normal testes and claims that his karyotype at birth showed 46, XY, he complains of sparse body hair and abnormal breast growth. Blood tests show an elevated testosterone:DHT ratio with normal testosterone levels. All other steroids were also at normal levels. Administration of what drug would induce a similar clinical presentation?

A. 5α-reductase inhibitor

B. Androgen receptor antagonist

C. CYP17 inhibitor

D. Glucocorticoid

E. GnRH antagonist

Use the following vignette for questions 8-10.

A 50-year-old trans male patient comes into the office for a routine visit to monitor his hormone levels. He has no past medical history of surgeries.

8. Prior to having started testosterone, which of the following hormone levels reflects changes in the late follicular phase compared to mid follicular phase? (↑,increased; ↓, decreased; N, no change) (E2=estrogen, FSH=follicle-stimulating hormone)

E2 FSH Inhibin B Activin

A. ↑ ↑ ↑ ↑

B. ↑ ↓ ↑ ↓

C. ↑ ↑ ↑ ↓

D. ↑ ↓ ↓ ↑

E. ↑ ↑ ↓ ↑

9. Once he began administering parenteral testosterone (the typical regimen for female-to-male therapy), it directly acts on various tissues. What physiological effects would be expected to occur within the first 6 months?

A. Fat redistribution

B. Hoarseness of voice

C. Typical cis male pattern baldness

D. Typical cis male pattern facial hair and beard

E. Vaginal enlargement

10. In addition to the physiological changes described in question 8, the patient reported the cessation of his menses. This effect is due to negative feedback of GnRH. What role does testosterone play in this mechanism?

A. Metabolism into androstenedione by CYP17 in granulosa cells

B. Metabolism into androstenedione by CYP17 in theca cells

*C. Metabolism into estrogen by CYP19 in granulosa cells

D. Metabolism into estrogen by CYP19 in theca cells

E. Metabolism into pregnenolone by CYP11A in granulosa cells

F. Metabolism into pregnenolone by CYP11A in theca cells

**C is the correct answer**. Testosterone will be metabolized by CYP19 (aromatase) into estrogen in granulosa cells. It also converts androstenedione into E_1_ (estrone). CYP17 metabolizes progesterone into androstenedione and pregnenolone into DHEA in theca cells, and cholesterol is converted by CYP11A into pregnenolone in theca and granulosa cells.

A is incorrect as androstenedione is converted by aromatase

B is incorrect as androstenedione is converted in granulosa cells

D is incorrect as estrogen is metabolized in granulosa cells

E is incorrect as pregnenolone is metabolized by CYP17

F is incorrect for a similar reason as E

*Additional Discussion Question: How do theca and granulosa cells produce their respective hormones?*

11. A 6-year-old boy is brought to his pediatrician due to the recent appearance of public and axillary hair. His mother reports a healthy pregnancy, and the child’s development and general health prior to the appearance of body hair was ‘normal’. Physical exam shows a well-developed phenotypical male child with descended testes. His vitals are all within normal ranges. Which of the following would best explain the most likely etiology of this boy’s symptoms?

A. Constitutively active FSH receptor

B. CYP17 loss-of function mutation

C. CYP19 gain-of-function mutation

*D. Gonadotropin-secreting pituitary adenoma

E. LH receptor loss-of-function mutation

**D is correct.** This child appears to have developed precocious puberty; that is, the premature activation of testicular androgen production. Normally, the age-appropriate awakening of the hypothalamic GnRH pulse generator triggers the secretion of the gonadotropins (LH and FSH) by the anterior pituitary gonadotropes, and this signals the onset of puberty.

A is incorrect as FSH activation would drive spermatogenesis in Sertoli cells, not testosterone production

B is incorrect as a loss-of-function in CYP17 would block testosterone synthesis causing the opposite effects of precocious puberty

C is incorrect as a gain-of-function in CYP19 (i.e. aromatase) would increase aromatization of androstenedione and testosterone into E2. This ultimately causes elevated E2 and decreased testosterone.

E is incorrect as a loss of LH function would block testosterone production by Leydig cells.

12. A 23-year-old woman presents to her gynecologist due to oligomenorrhea over the past year (periods occurring approximately every 35-40 days, with some missed periods), the appearance of facial acne, and facial hair over her upper lip. Her prior medical history is unremarkable, she has never been pregnant. Physical exam shows a well-developed female, BMI = 26 kg/m^2^. Vitals include BP of 140/65 mm Hg, pulse 70/min, temp 37 °C, SpO_2_ 99%. If measured and relative to normal, which of the following blood lab panels would most likely be obtained in this woman? (↑,increased; ↓, decreased; N, no change) (SHBG, sex hormone binding globulin; HDL, high density lipoprotein; FSH, follicle-stimulating hormone)

SHBG FSH Free testosterone HDL

A. ↑ ↑ ↑ ↑

B. ↑ ↓ ↓ ↑

*C. ↓ ↓ ↑ ↓

D. ↓ ↑ ↑ ↓

E. N ↓ ↓ N

F. N ↑ ↓ N

*Additional Discussion Question: what are treatment options for polycystic ovary syndrome?*

13. During fetal development, either the Müllerian duct or the Wolffian duct will not regress and progress into gonads. In biological males, SRY and TDF stimulate the testis to secrete testosterone and AMH. What will happen to the ducts?

A. Müllerian duct becomes internal gonads, and Wolffian duct becomes external gonads.

B. Müllerian duct becomes internal gonads, and Wolffian duct regresses.

C. Wolffian duct and Müllerian duct become internal gonads.

D. Wolffian duct becomes internal gonads, and Müllerian duct becomes external gonads.

E. Wolffian duct becomes internal gonads, and Müllerian duct regresses.

*Additional Discussion Question: What do the Wolffian and Müllerian ducts become, respectively, in adults? What is the effect of DHT on genital development?*

14. Your patient, a trans man, and his partner are in the process of starting a family. Having temporarily stopped testosterone more than 6 months prior to his pregnancy, he is currently 25 weeks pregnant. Which of the following describes the effects of E_2_ and progesterone on the following systems?

Na+ reabsorption Water reabsorption Minute ventilation

A. ↑ ↑ ↓

B. ↑ ↑ ↓

C. ↑ ↑ ↑

D. ↓ ↓ ↓

E. ↓ ↓ ↑

*Additional Discussion Question: Why is increased Na+ and water reabsorption, increased minute ventilation, and vasodilation important during pregnancy?*

15. Your patient from question 14 is now 35 weeks pregnant, and you measure his hormone levels to check on his progress. Which of the following hormone profiles would you expect to see that will prepare the uterus for delivery? (P_4_ = progesterone, E_3_ = estriol, E_2_ = estradiol-17β)

A. E_2_ > E_3_

B. E_3_ > P_4_

C. E_3_ > E_2_

D. P_4_ > E_2_

E. P_4_ > E_3_

*Additional Discussion Question: What effect do oxytocin, prostaglandins, and relaxin play during labor and delivery?*

**Case Discussion**

**Please note that you only have the answer to the fourth question provided below**

Use the discussion points to guide a discussion within your group. If you have outside resources from the previous section that are helpful during these discussions, please feel free to use and share them.

You knock and walk into the exam room to see your next patient, who is a new patient. Upon first impression, the patient appears to be male-presenting and generally well although fidgety and tense while sitting in the chair. How would you start the conversation? (Think PCM)

The patient states that her legal name is John Smith but has recently starting going by Diane. She is 32 and uses she/her pronouns. When you ask what brings her to the clinic, she explains that she wants to start gender-affirming hormone therapy. What additional information would you want to know from the patient regarding hormone therapy?

Diane explains that she has researched some things on the internet and tried to go to her previous primary care physician to start hormone therapy, but they had no experience in the area. How would you explain hormone therapy to Diane? Be sure to include what physiologic effects would NOT be seen and adverse effects

After hearing your explanation, Diane still wants to start therapy. You move on to complete the past medical history and rest of the clinical interview. What specific information would you not want to miss in each of the sections?

Past Medical History:

*History of DVTs, thrombosis, atherosclerosis*

*History of HTN, diabetes, dyslipidemia, liver disease*

*History of mental illness*

Past Surgical History:

*Removal of any gonads (e.g., orchiectomy), cosmetic surgeries*

*Appendix, gall bladder, and tonsils are often surgeries that occur early in life, so people can forget that these occurred*

Family History:

*History of DVTs, thrombosis, atherosclerosis*

*History of HTN, diabetes, dyslipidemia*

Medications:

*Any medications for the above diseases*

*Any over-the-counter medications*

*Herbal remedies*

Allergies:

*Any allergies and what happens with the allergy*

Other:

*Last time seen by a PCP*

*Key Points:*

- *Make sure the patient is generally healthy as this is a medical intervention, and you will be starting a new drug*
  - *Remember estrogen causes increased risk of venous thromboembolism, so any history or family history of clots warrants extra investigation and counseling*
- *Members of the LGBTQ+ community tend to have higher rates of mental illnesses including Major Depressive Disorder and Substance Use Disorder*
- *Important: trans people DO NOT all have Gender Dysphoria.*
  - *Gender Dysphoria (GD), as defined by the DSM-V, is “a marked incongruence between one’s experienced/expressed gender, of at least 6 months’ duration” resulting in “clinically significant distress or impairment in social, occupational, or other important areas of functioning.” Insurance companies, however, may require a diagnosis of GD to qualify for coverage.*
- *Trans patients are less likely to seek healthcare: faced prior discrimination, providers weren’t knowledgeable or interested in learning more, institutional barriers like non-inclusive health forms, continuous misnaming*
- *Must ask about what organs patients still have to determine screening for cancers (e.g., trans man can be on hormones but still have all sex organs intact and will need to be screened for cervical cancer)*

You then take a detailed Social History. What information would you want to know?

Social History:

Sexual history:

You finish taking a full history of Diane. You then explain the typical treatment regimen for a male-to-female transition. After getting the results for an initial health screen, you will start her on low doses of oral estradiol and oral spironolactone. She will have to come in every three months to measure blood hormone levels. The goal will be to lower testosterone levels to the female range. When she comes in for her checkups, what parameters should be measured?

At the conclusion of her exam, she mentions that she would like some feminization surgeries in the future. You counsel her on various options including facial feminization surgeries, breast augmentation, and genital reconstruction surgeries like vaginoplasty and orchiectomy (removal of the testes). How do surgeries affect screening practices? Consider both transmen and transwomen.
