# Supplementary material for: Teaching Trans-Centric Curricular Content Using Modified Jigsaw
Source: MedEdPORTAL. 2022 May 24;18:11257. doi: 10.15766/mep_2374-8265.11257 (PMC9127030; doi:10.15766/mep_2374-8265.11257)
Supplement: Supplementary file 1 — Activity and Materials Outline.docxFacilitator Guide.docxPresession Survey.docxPretest Questions.docxStudent Packet 1.docxStudent Packet 2.docxStudent Packet 3.docxStudent Packet 4.docxStudent Packet 5.docxSimulated Transgender Patient Interview.mp4Posttest Questions.docxPosttest Answers.docxPostsession Survey.docx [file mep_2374-8265.11257-s001.zip › A. Activity and Materials Outline.docx]

**Authors’ note**: this session was designed in late 2019. Language and approaches to discussing the transgender community evolve and change with time. As such, aspects of this content may need to be updated prior to implementation. Resources to help in updating include

- https://www.hrc.org/resources/glossary-of-terms
- https://pflag.org/glossary
- https://thesafezoneproject.com/resources/vocabulary/
- https://www.ama-assn.org/about/ama-center-health-equity/advancing-health-equity-guide-language-narrative-and-concepts-0

**Table of Contents**

Appendix A – Activity Outline: instruction packet for facilitating activity. Contains table of contents for appendices and session timeline

Appendix B – Facilitator Guide: full answers and discussion points of pre-test, case discussion, and video discussion. Also includes notes regarding content facilitators should prepare for. To be given to facilitators ahead of session.

Appendix C – Pre-session survey: assesses students’ prior experiences with the trans community, attitudes, perceptions, and self-confidence relating to trans medicine topics at the beginning of the session

Appendix D – Pre-test: 15 knowledge questions (5 specifically tied to transgender medicine) for students to answer individually at the beginning of the session

Appendix E-I – Participant Packets 1-5: all answers for pre-test and discussion points for case discussion will be divided amongst the 5 packets. Each student should have answers to 3 pre-test questions and discussion points for 1-2 discussion questions. Each group should have 1 of each packet for complete set of answers. In the event a group does not have exactly 5 students, students may need to be responsible for 2 packets.

Appendix J – Simulated Transgender Patient Interview: video for large group discussion. Details encounter a trans male presenting for initial evaluation of abdominal pain. Discussion points for this video are in Appendix B

Appendix K – Post-test Questions: 5 question test of transgender medicine only. To be used at end of session for all students to complete.

Appendix L – Post-test Answers: post-test questions with answers only (no explanations). Can be distributed to students after completion of session for review.

Appendix M – Post-session Survey: post-session survey questions only. Contains similar questions as pre-session survey and assesses attitudes, perceptions, and self-confidence relating to trans medicine topics. To be distributed at the end of the session

**Suggested Topics for Students to be Familiar with**

As this session was designed to be nested in existing endocrine and reproduction physiology courses, students should have been exposed (e.g., obtained a lecture, completed a self-directed learning activity) to the following topics:

- Male reproductive physiology and anatomy: synthesis of testosterone, embryology and development of testes, physiologic effects of testosterone and DHT, function of Sertoli and Leydig cells
- Female reproductive physiology and anatomy: synthesis of estrogen and progesterone, embryology and development of ova, physiologic effects of estrogen and progesterone, menstrual cycle, pregnancy physiology, function of granulosa and theca cells
- Hypothalamic-pituitary-gonadal axis: activators and inhibitors, feedback loops
- Commonly used drugs in managing reproductive diseases including, but not limited to: androgen receptor inhibitors, 5a-reductase inhibitors, CYP17 inhibitors, aldosterone receptor inhibitors, glucocorticoids, GnRH agonists, GnRH antagonists, aromatase inhibitors

Students should also have some knowledge of clinical skills specifically:

- Basic interviewing technique: introductions (e.g., Hi, my name is…and I am a first-year medical student at…), structure and contents of a standard history, eliciting patient understanding

Students do not need to have extensive experience practicing their skills in a true clinical environment (e.g., working with a physician at a clinic), however, some experience with simulated patients would be helpful

We implemented this session at the end of the endocrine-reproduction course a few days prior to the final exam in the beginning of the 2^nd^ semester for first-year students. We found that students had an adequate fund of knowledge in physiology concepts as well as clinical interviewing.

**Session Structure and Groups**

- Students divided into **groups of 5**
  - If in person, ideally choose room that has ample space to allow for students to huddle together
  - If online, using a breakout room feature for each group allows for uninterrupted conversations among group members

Students will stay in this group throughout the duration of the activity

**Session Schematic**

This schematic shows timeline if one clinical correlation activity (i.e. the case discussion or the video discussion) is implemented. This format is what was used to pilot the modified jigsaw (case discussion in 2020 and group video discussion in 2021).

Pre-session survey and pretest with discussion

(1 hour 25 minutes)

21-question survey

15-question test:

physiology and transgender medicine

Groups of 4-5:

Each student has answers to 2-3 questions and teaches group

Post-test and post-session survey

(15 minutes)

6-question test: trans medicine only

15-question survey

Group video discussion:

(30 minutes)

Watch simulated encounter between trans patient and provider

Facilitator panel discussion of patient encounter

Case discussion:

(30 minutes)

Small group discussion of clinical encounter with trans patient

Each student leads 1-2 discussion questions

**Ideal Session Timeline**

This timeline shows an ideal timeline where the case discussion **and** video discussion are both completed. Notes and activity descriptions are applicable for all iterations of the activity. See Appendix B for more detail regarding each activity.

| **Time Elapsed** | **Activity** | **Other Notes** |
| --- | --- | --- |
| 5 minutes | Students are getting settled. Encourage them to begin the individual survey and pre-test (Appendix C, D) as soon as possible | Students are divided into groups of 5 (seated at tables in-person or divided into breakout rooms virtually)  Group assignments should be distributed prior to arriving (e.g., online portal, email). |
| 40 minutes | Students should be taking the pre-survey and pre-test. There are 22 survey questions and 15 test questions. | Pre-test can be distributed on paper (if in person) or online.  If students finish early, encourage them to begin preparing their answers for the discussion. |
| 40 minutes | Students should begin group discussion using student packets 1-5 (Appendix E-I) | Facilitators should circle room to answer any questions.  If students finish early, encourage them to read ahead and prepare answers for case discussion individually  ****Most likely area to run over time as students may not finish in 40 minutes**** |
| 12 minutes | Reconvene as group to watch interview video (Appendix J) | Either share with entire room if in-person or call students back from breakout rooms if virtual |
| 18 minutes | Facilitator-led whole room discussion of simulated encounter (Discussion points in Appendix B) | Facilitators should answer any questions students have |
| 20 minutes | Simulated case discussion using student packets 1-5 (Appendix E-I) | Facilitators should circle room and answer any questions |
| 15 minutes | There are 23 survey questions and 6 test questions (Appendix K, M) | Post-test and post-survey should be distributed at this time |
| **150 minutes total** |  |  |
