# Supplementary material for: Teaching Trans-Centric Curricular Content Using Modified Jigsaw
Source: MedEdPORTAL. 2022 May 24;18:11257. doi: 10.15766/mep_2374-8265.11257 (PMC9127030; doi:10.15766/mep_2374-8265.11257)
Supplement: Supplementary file 1 — Activity and Materials Outline.docxFacilitator Guide.docxPresession Survey.docxPretest Questions.docxStudent Packet 1.docxStudent Packet 2.docxStudent Packet 3.docxStudent Packet 4.docxStudent Packet 5.docxSimulated Transgender Patient Interview.mp4Posttest Questions.docxPosttest Answers.docxPostsession Survey.docx [file mep_2374-8265.11257-s001.zip › B. Facilitator Guide.docx]

**General Concepts that will be Discussed**

- Male reproductive physiology: synthesis of testosterone, physiologic effects of testosterone
- Female reproductive physiology: synthesis of estrogen and progesterone, physiologic effects of progesterone and estrogen
- Gender-affirming hormone therapy: discussing treatment in context of patient’s wants, anticipated physiologic effects of feminizing/masculinizing regimens
- Disparities of transgender community: substance use, access to healthcare, mental health disorders, lack of social support
- Feminizing and masculinizing surgery options
- Personal clinical experiences in treating trans patients including any difficulties noted, socioeconomic barriers that needed to be navigated, learning points

**Question Distribution Key**

The following student packets have answers to these questions

Packet 1: Pretest Q1, 8, 12. Discussion: introduction, eliciting understanding of hormone therapy

Packet 2: Pretest Q2, 3, 7. Discussion: explanation of hormone therapy
Packet 3: Pretest Q4, 10, 11. Discussion: eliciting clinical interview

Packet 4: Pretest Q5, 6, 13. Discussion: eliciting social and sexual history

Packet 5: Pretest Q9, 14, 15. Discussion: monitoring of hormone therapy, general health screening

**Pre-test Questions**

Use the following vignette for questions 1-4.

A 25-year-old masculine-appearing patient goes to the primary care physician to discuss starting hormone therapy. Assigned male at birth, she states that she has never identified being “male” even as a child and instead identifies as female. As a first step, she recently started going by a different name and using “she/her” as her pronouns. She finds her physical appearance particularly distressing and would like to feminize her features.

1. What is the most appropriate term to describe the gender identity of this patient?

A. Cisgender

B. Gay

C. Genderqueer

*D. Transgender

E. Transvestite

**D is correct**. Transgender is currently the most accepted form of describing an individual whose gender identity is different from the sex assigned at birth.

A is incorrect as cisgender refers to individuals who identify with their assigned sex.

B is incorrect as “gay” describes a sexual orientation, which refers to whom the individual is attracted to. Gender identity refers to an individual’s internal sense of where they are on the gender spectrum.

C is incorrect as it typically refers to individuals who feel that are both male and female or neither. Since the patient currently identifies as female, it would not be the best option. However of note, the best way to determine how your patient identifies is to directly ask.

E is incorrect as “transvestite” is largely outdated and considered offensive by many in the LGBTQ+ community. Cross-dresser has replaced the term to typically refer to a male who occasionally wears clothes, makeup, accessories that are associated with females as a form of gender identity.

After a thorough examination and discussion, the physician starts her on a feminizing hormone regimen.

2. What would be the best drugs to use in this scenario? (Answers listed as primary drug and adjuvant)

A. Finasteride and spironolactone

B. Oral estradiol and cortisol

C. Oral estradiol and progestins

*D. Oral estradiol and spironolactone

E. Spironolactone and progestins

**D is correct.** The typical regimen involves giving direct estradiol and then an adjuvant to lower androgens, in this case spironolactone. If you were to give estradiol alone, you would require very high amounts to suppress testosterone.

A is incorrect since both finasteride and spironolactone will inhibit the release of testosterone (finasteride through inhibition of 5-α-reductase-2, and spironolactone through inhibition of androgen receptors). There is no source of estrogen.

B is incorrect because although cortisol will lower testosterone levels, prolonged use of steroids results in numerous undesirable side effects.

C is incorrect. While you do have oral estradiol, which will serve as an exogenous source of hormones, progestins are not recommended for use. Although they exert negative feedback on the GnRH axis, their high risk of cardiovascular and breast cancer make them less desirable.

E is incorrect for similar reasons that A is incorrect: no exogenous source of estradiol.

*Additional Discussion Question: what are the side effects of prolonged steroid use?*

3. What is a mechanism by which the adjuvant drug acts?

A. Central suppression of GnRH

B. Increasing release of prolactin

C. Inhibition of 5α-reductase

*D. Androgen receptor antagonist

E. Stimulation of Sertoli cells to secrete more inhibin B

*Additional Discussion Question: what is the synthesis pathway of testosterone?*

**D is correct.** From the previous question, knowing that the adjuvant is likely spironolactone, its method of action in regards to androgens is as an androgen receptor antagonist. It also has effects in reducing overall testosterone levels.

A is incorrect and is a characteristic of GnRH antagonists or chronic GnRH agonist use.

B is incorrect. While increased prolactin would inhibit GnRH, spironolactone has no effect on it.

C is incorrect and is a characteristic of 5a-reductase inhibitors such as finasteride

E is incorrect as spironolactone has no direct effect on Sertoli cells

4. Through the prescribed treatment regimen what physiological changes would you expect to occur?

A. Changes in bone shape

*B. Changes in skin

C. Increased libido

D. Increased pitch of voice

E. Increased red blood cells

**B is correct**. Estrogen will induce effects including decreased oiliness and softening of skin, breath tissue growth, redistribution of fat, and decreased facial and body hair.

A is incorrect as estrogen will not reverse the prior effects of androgens on bone structure.

C is incorrect. Patients will in fact report reduce libido.

D is incorrect for a reason similar to B. Estrogen has no effect on already formed cartilage structures. As this patient is an adult, bones will not be actively growing.

E is incorrect. That is an important side effect of testosterone treatment.

5. Which drug is incorrectly matched to its side effect?

A. Cyproterone – hyperprolactinemia

B. Estrogen – increased risk of venous thromboembolism

*C. Finasteride - hypercalcemia

D. Spironolactone – hyperkalemia

E. Testosterone – polycythemia

**C is correct**. Finasteride does not have a risk of hypercalcemia.

A is incorrect as cyproterone can cause hyperprolactinemia

B is incorrect as estrogen causes increased risk of VTE

D is incorrect as spironolactone causes hyperkalemia

E is incorrect as testosterone causes polycythemia

*Additional Discussion Question: what tests could you do to monitor the side effects of these drugs? (e.g., how would you measure hyperkalemia?)*

6. _____ stimulates _______ which in turn secrete androgen binding protein and inhibin B.

A. FSH; Leydig cells

*B. FSH; Sertoli cells

C. LH; Leydig cells

D. LH; Sertoli cells

E. GnRH; pituitary

**B is correct**. GnRH stimulates the pituitary to release FSH and LH. FSH will then stimulate Sertoli cells to secrete factors including inhibin B, androgen binding protein, and nutrient binding proteins. LH stimulates Leydig cells to metabolize cholesterol into testosterone and subsequently secrete it.

A is incorrect as FSH stimulates Sertoli cells

C is incorrect as Leydig cells do not secrete androgen binding protein or inhibin B

D is incorrect as LH stimulates Leydig cells

E is incorrect as GnRH and the pituitary do not secrete androgen binding protein or inhibin B

*Additional Discussion Question: what do inhibin B and androgen binding protein do?*

7. A 16-year-old male-appearing patient goes to the endocrinologist due to an “abnormal” puberty. While he has normal testes and claims that his karyotype at birth showed 46, XY, he complains of sparse body hair and abnormal breast growth. Blood tests show an elevated testosterone:DHT ratio with normal testosterone levels. All other steroids were also at normal levels. Administration of what drug would induce a similar clinical presentation?

*A. 5α-reductase inhibitor

B. Androgen receptor antagonist

C. CYP17 inhibitor

D. Glucocorticoid

E. GnRH antagonist

**A is the correct answer**. Based on the vignette, the patient presents with 5α-reductase deficiency. They will physically present with relatively mild symptoms in female and symptoms as described above in males. These patients are able to produce testosterone and other steroid hormones but lack the ability to convert testosterone to DHT.

B is incorrect. Similar physical symptoms would be produced, however, blood tests would show decreased levels for multiple steroids for CYP17 deficiency.

C is incorrect. Like B, similar physical symptoms would be produced, but blood tests would show abnormal levels for multiple steroids for androgen insensitivity.

D is incorrect as excess cortisol (i.e. Cushing’s syndrome) in the body would have metabolic, immune, and musculoskeletal symptoms, to name a few affected systems.

E is incorrect. Although physical presentation may be similar, the blood workup would show deficiencies in multiple steroid hormones.

Use the following vignette for questions 8-10.

A 50-year-old trans male patient comes into the office for a routine visit to monitor his hormone levels. He has no past medical history of surgeries.

8. Prior to having started testosterone, which of the following hormone levels reflects changes in the late follicular phase compared to mid follicular phase? (↑,increased; ↓, decreased; N, no change) (E2=estrogen, FSH=follicle-stimulating hormone)

E2 FSH Inhibin B Activin

A. ↑ ↑ ↑ ↑

B. ↑ ↓ ↑ ↓

C. ↑ ↑ ↑ ↓

D. ↑ ↓ ↓ ↑

*E. ↑ ↑ ↓ ↑

**E is the correct answer**. Early in the follicular phase, E2 exerts negative feedback on the GnRH pulse generator, and preovulatory granulosa cells produce inhibin B which inhibits activin. Towards the latter half of the follicular phase, E2 levels increase to a point that it now exerts positive feedback on FSH secretion. Inhibin B levels also drop and allow activin to further stimulate FSH secretion.

A is incorrect as inhibin B levels would drop

B is incorrect as FSH increases due to positive feedback from E2, and inhibin B decreases

C is incorrect as activin increases due to decreased inhibin B

D is incorrect as FSH increases

9. Once he began administering parenteral testosterone (the typical regimen for female-to-male therapy), it directly acts on various tissues. What physiological effects would be expected to occur within the first 6 months?

*A. Fat redistribution

B. Hoarseness of voice

C. Typical cis male pattern baldness

D. Typical cis male pattern facial hair and beard

E. Vaginal enlargement

**A is the correct answer**. Fat redistribution is one of the changes seen in approximately 1-6 months. Patients also report increased acne and deepening of voices. Height is unchanged for reasons similar to those in female-to-male hormone therapy.

B is incorrect as voice hoarseness does not occur though deepening will

C is incorrect as changes in hair typically take longer than 6 months to occur

D is incorrect for similar reasons as C

E is incorrect as the vagina does not change size. The clitoris, however, may increase in size

10. In addition to the physiological changes described in question 8, the patient reported the cessation of his menses. This effect is due to negative feedback of GnRH. What role does testosterone play in this mechanism?

A. Metabolism into androstenedione by CYP17 in granulosa cells

B. Metabolism into androstenedione by CYP17 in theca cells

*C. Metabolism into estrogen by CYP19 in granulosa cells

D. Metabolism into estrogen by CYP19 in theca cells

E. Metabolism into pregnenolone by CYP11A in granulosa cells

F. Metabolism into pregnenolone by CYP11A in theca cells

**C is the correct answer**. Testosterone will be metabolized by CYP19 (aromatase) into estrogen in granulosa cells. It also converts androstenedione into E_1_ (estrone). CYP17 metabolizes progesterone into androstenedione and pregnenolone into DHEA in theca cells, and cholesterol is converted by CYP11A into pregnenolone in theca and granulosa cells.

A is incorrect as androstenedione is converted by aromatase

B is incorrect as androstenedione is converted in granulosa cells

D is incorrect as estrogen is metabolized in granulosa cells

E is incorrect as pregnenolone is metabolized by CYP17

F is incorrect for a similar reason as E

*Additional Discussion Question: How do theca and granulosa cells produce their respective hormones?*

11. A 6-year-old boy is brought to his pediatrician due to the recent appearance of public and axillary hair. His mother reports a healthy pregnancy, and the child’s development and general health prior to the appearance of body hair was ‘normal’. Physical exam shows a well-developed phenotypical male child with descended testes. His vitals are all within normal ranges. Which of the following would best explain the most likely etiology of this boy’s symptoms?

A. Constitutively active FSH receptor

B. CYP17 loss-of function mutation

C. CYP19 gain-of-function mutation

*D. Gonadotropin-secreting pituitary adenoma

E. LH receptor loss-of-function mutation

**D is correct.** This child appears to have developed precocious puberty; that is, the premature activation of testicular androgen production. Normally, the age-appropriate awakening of the hypothalamic GnRH pulse generator triggers the secretion of the gonadotropins (LH and FSH) by the anterior pituitary gonadotropes, and this signals the onset of puberty.

A is incorrect as FSH activation would drive spermatogenesis in Sertoli cells, not testosterone production

B is incorrect as a loss-of-function in CYP17 would block testosterone synthesis causing the opposite effects of precocious puberty

C is incorrect as a gain-of-function in CYP19 (i.e. aromatase) would increase aromatization of androstenedione and testosterone into E2. This ultimately causes elevated E2 and decreased testosterone.

E is incorrect as a loss of LH function would block testosterone production by Leydig cells.

12. A 23-year-old woman presents to her gynecologist due to oligomenorrhea over the past year (periods occurring approximately every 35-40 days, with some missed periods), the appearance of facial acne, and facial hair over her upper lip. Her prior medical history is unremarkable, she has never been pregnant. Physical exam shows a well-developed female, BMI = 26 kg/m^2^. Vitals include BP of 140/65 mm Hg, pulse 70/min, temp 37 °C, SpO_2_ 99%. If measured and relative to normal, which of the following blood lab panels would most likely be obtained in this woman? (↑,increased; ↓, decreased; N, no change) (SHBG, sex hormone binding globulin; HDL, high density lipoprotein; FSH, follicle-stimulating hormone)

SHBG FSH Free testosterone HDL

A. ↑ ↑ ↑ ↑

B. ↑ ↓ ↓ ↑

*C. ↓ ↓ ↑ ↓

D. ↓ ↑ ↑ ↓

E. N ↓ ↓ N

F. N ↑ ↓ N

**C is correct.** Her symptoms of recent onset acne and hirsutism with oligomenorrhea suggest androgen excess. This makes choices B, E, F less likely. Elevated testosterone decreases the production of the androgen binding protein SHBG, enabling higher levels of serum free testosterone. Testosterone exerts negative feedback on FSH (and LH) secretion, which is disrupting the normal neuroendocrine control of ovarian function, thus impacting her menstrual cycle. Finally, elevated testosterone has the potential to lower HDL levels. Based upon her BMI, she appears to be overweight; more testing should investigate the possibility that this woman has developed polycystic ovary syndrome.

A is incorrect as SHGB production would be inhibited by testosterone. FSH would also decrease, and HDL would be lowered.

B is incorrect as SHBG production is inhibited, and free testosterone would be increased

D is incorrect as FSH is inhibited through negative feedback

E is incorrect as SHBG and HDL are affected and free testosterone would increase.

F is incorrect as SHBG and HDL are affected, free testosterone would increase, and FSH would decrease.

*Additional Discussion Question: what are treatment options for polycystic ovary syndrome?*

13. During fetal development, either the Müllerian duct or the Wolffian duct will not regress and progress into gonads. In biological males, SRY and TDF stimulate the testis to secrete testosterone and AMH. What will happen to the ducts?

A. Müllerian duct becomes internal gonads, and Wolffian duct becomes external gonads.

B. Müllerian duct becomes internal gonads, and Wolffian duct regresses.

C. Wolffian duct and Müllerian duct become internal gonads.

D. Wolffian duct becomes internal gonads, and Müllerian duct becomes external gonads.

*E. Wolffian duct becomes internal gonads, and Müllerian duct regresses.

**E is the correct answer**. Testosterone stimulates the Wolffian duct to develop. AMH, anti-Müllerian hormone, causes the Müllerian duct to regress. In females, a lack of testosterone and AMH causes the Wolffian duct to regress.

A is incorrect as Müllerian ducts regress in biological males

B is incorrect for similar reasons as A

C is incorrect for similar reasons as A

D is incorrect for similar reasons as A

*Additional Discussion Question: What do the Wolffian and Müllerian ducts become, respectively, in adults? What is the effect of DHT on genital development?*

14. Your patient, a trans man, and his partner are in the process of starting a family. Having temporarily stopped testosterone more than 6 months prior to his pregnancy, he is currently 25 weeks pregnant. Which of the following describes the effects of E_2_ and progesterone on the following systems?

Na+ reabsorption Water reabsorption Minute ventilation

A. ↑ ↑ ↓

B. ↑ ↑ ↓

*C. ↑ ↑ ↑

D. ↓ ↓ ↓

E. ↓ ↓ ↑

**C is the correct answer**. E_2_ stimulates the renin-angiotensin system, which stimulates increased sodium and water reabsorption. Progesterone and E_2_ also stimulate AVP, arginine vasopressin, which results in further water reabsorption. Progesterone stimulates medullary respiratory centers and carotid body chemoreceptors to increase ventilatory drive and minute ventilation. To note, there have been some studies done examining transgender men during pregnancy, delivery, and birth outcomes. No conclusive evidence has yet been found to indicate adverse outcomes correlated with prior testosterone use.

A is incorrect as minute ventilation and venilatory drive will increase due to progesterone stimulating medullary respiratory centers and carotid body chemoreceptors

B is incorrect as water reabsorption would increase due to E_2_ stimulating RAS, and progesterone and E2 stimulating AVP.

D is incorrect for similar reasons as A and B. Na+ reabsorption would also be increased due to RAS and AVP activation

E is incorrect for similar reasons as D.

*Additional Discussion Question: Why is increased Na+ and water reabsorption, increased minute ventilation, and vasodilation important during pregnancy?*

15. Your patient from question 14 is now 35 weeks pregnant, and you measure his hormone levels to check on his progress. Which of the following hormone profiles would you expect to see that will prepare the uterus for delivery? (P_4_ = progesterone, E_3_ = estriol, E_2_ = estradiol-17β)

A. E_2_ > E_3_

B. E_3_ > P_4_

*C. E_3_ > E_2_

D. P_4_ > E_2_

E. P_4_ > E_3_

**C is the correct answer**. Throughout the pregnancy, E_3_ and E_2_ levels remain relatively similar until the third trimester where there will be almost a 10-fold increase in E_3_ levels due to an increase in DHEA levels (mostly from the fetal adrenal glands). This activates the placental ER to begin expression of myometrial contractile proteins.

A is incorrect as E_3_ will drastically increase compared to E_2_

B is incorrect as P_4_ is present in higher levels than E_3_

D is incorrect because P_4_ does not play a role in preparing for contractions, instead exerting a quiescing effect within myometrium. It is correct that P_4_ is present in higher levels than both E_3_ and E_2_

E is incorrect for similar reasons as D

*Additional Discussion Question: What effect do oxytocin, prostaglandins, and relaxin play during labor and delivery?*

**Case Discussion**

You knock and walk into the exam room to see your next patient, who is a new patient. Upon first impression, the patient appears to be masculine-presenting and generally well although fidgety and tense while sitting in the chair. How would you start the conversation?

*Hi, my name is XYZ and I’m a first-year medical student ABC Medical School. Can I have you confirm your name and age for me? And how would you like to be addressed? My pronouns are XYZ; what are your pronouns?*

*Key points:*

- *Confirmation of legal name*
- *Name that patient goes by and make note of that on patient chart*
- *Pronouns: avoid saying “preferred” as it isn’t merely a preference*
- *If you’re ever unsure about something, ask (for example, although this patient is masculine-presenting, you should not assume the patient uses he/him pronouns)*

The patient states that her legal name is John Smith but has recently started going by Diane. She is 32 and uses she/her pronouns. When you ask what brings her to the clinic, she explains that she wants to start gender-affirming hormone therapy. What additional information would you want to know from the patient regarding hormone therapy?

- *Patient’s understanding of what hormone therapy is: effects, risks, benefits*
- *Patient expectations of effects (magnitude of effects, how fast effects are expected to be seen): for example, if the patient wants to start masculinizing hormone therapy, do they expect to immediately begin growing facial hair or grow an Adam’s apple?*
- *Patient’s goals for hormone therapy: what kind of physical effects do they want to see?*
- *How long they have considered hormone therapy*
- *Prior discussions about hormone therapy: have they spoke about it friends, other members of the trans community, other providers, etc.?*
- *Prior experience with hormones: previous provider, personal purchase of hormones, etc.*

Diane explains that she has researched some things on the internet and tried to go to her previous primary care physician to start hormone therapy, but they had no experience in the area. How would you explain hormone therapy to Diane? Be sure to include what physiologic effects would NOT be seen and adverse effects

*Example answer: Your body naturally produces testosterone, which produces a lot of the physical effects we associate with masculinity like increased muscle tone, increased facial and body hair, and deeper voice. To align your physical appearance more with your gender identity, we will have to lower your testosterone levels and give you estrogen, which will produce more feminized features such as breast growth, fat redistribution, and smoother skin. You may also feel a decreased sex drive, as that is related to testosterone. Gender-affirming hormone therapy WILL NOT change the structure of your bones or lower your voice. There is likely an increased risk in venous thromboembolic disease, whereby you’ll be more likely to form clots in your veins that can travel to other parts of your body. Other side effects can include increased triglycerides and hypertension. There may be decreased fertility, so you may want to consider family planning options before starting therapy.*

*Key points*

- *Explanation of testosterone and its physiologic effects*
- *Explanation of how male-to-female hormone therapy aims to lower testosterone levels and raise estrogen levels*
- *Explanation of exogenous estrogen and its physiologic effects*
- *Effects that will not be seen with feminizing hormone therapy: changes in bone shape/size (e.g., Adam’s apple will remain), changes in voice (trans women will often go through voice coaching to learn how to voluntarily control their voices to match their self-image)*
- *Emphasize that not enough data exist showing the long-term effects of hormone therapy, but it seems relatively well-tolerated*

After hearing your explanation and confirming that she understands what you have discussed, Diane still wants to start therapy. You move on to complete the past medical history and rest of the clinical interview. What specific information would you not want to miss in each of the sections?

Past Medical History:

*History of DVTs, thrombosis, atherosclerosis*

*History of HTN, diabetes, dyslipidemia, liver disease*

*History of mental illness*

Past Surgical History:

*Removal of any gonads (e.g., orchiectomy), cosmetic surgeries*

*Appendix, gall bladder, and tonsils are often surgeries that occur early in life, so people can forget that these occurred*

Family History:

*History of DVTs, thrombosis, atherosclerosis*

*History of HTN, diabetes, dyslipidemia*

Medications:

*Any medications for the above diseases*

*Any over-the-counter medications*

*Herbal remedies*

Allergies:

*Any allergies and what happens with the allergy*

Other:

*Last time seen by a PCP*

*Key Points:*

- *Make sure the patient is generally healthy as this is a medical intervention, and you will be starting a new drug*
  - *Remember estrogen causes increased risk of venous thromboembolism, so any history or family history of clots warrants extra investigation and counseling*
- *Members of the LGBTQ+ community tend to have higher rates of mental illnesses including Major Depressive Disorder and Substance Use Disorder*
- *Important: trans people DO NOT all have Gender Dysphoria.*
  - *Gender Dysphoria (GD), as defined by the DSM-V, is “a marked incongruence between one’s experienced/expressed gender, of at least 6 months’ duration” resulting in “clinically significant distress or impairment in social, occupational, or other important areas of functioning.” Insurance companies, however, may require a diagnosis of GD to qualify for coverage.*
- *Trans patients are less likely to seek healthcare: faced prior discrimination, providers weren’t knowledgeable or interested in learning more, institutional barriers like non-inclusive health forms, continuous misnaming*
- *Must ask about what organs patients still have to determine screening for cancers (e.g., trans man can be on hormones but still have all sex organs intact and will need to be screened for cervical cancer)*

You then take a detailed Social History. What information would you want to know?

Social History:

*Smoking**

*Alcohol use*

*Other recreational drug use*

*Vaping*

*Activities that produce self-harm (cutting, burning)*

*Social support: “coming out,” family/friends*

*Do they know other individuals in the LGBTQ+ community?*

*Does patient have a permanent home or is in transient one or homeless*

*If in a home, do they feel safe?*

*Living alone or with others**?*

*Occupation/does this produce adequate income to avoid food insecurity, pay for medications, etc.*

Sexual history:

*Partner (sex, number of partners), forms of protection, PrEP and PEP*

*Family building considerations*

*Key Points:*

- *Smoking must be stopped prior to starting estrogen since it increases the risk of VTE*
- *Important to gauge social support since transitioning can be difficult: trans individuals experience higher rates of violence, harassment, and family rejection*
- *Also important to know if they are safe at home since LGBTQ+ individuals are at higher risk for being victims of interpersonal violence*
- *When asking about sexual partners, don’t assume gender of partner(s)*
  - *Remember, transgender is a gender identity, not a sexuality*
- *Important to discuss safe sex and PrEP and PEP to reduce risk of HIV: trans individuals have higher rates of STIs especially HIV*
- *Family building considerations should be discussed early since hormones may reduce fertility*
- ****Ask the group what it means to transition. Has Diane started this process?*
  - *While many may think that taking hormones or undergoing surgery is transitioning, the process starts much sooner. Social transitioning, such as changing name, pronouns, or clothing, is an important part of the process as well!****

You finish taking a full history of Diane. You then explain the typical treatment regimen for a male-to-female transition. After getting the results for an initial health screen, you will start her on low doses of oral estradiol and oral spironolactone. She will have to come in every three months to measure blood hormone levels. The goal will be to lower testosterone levels to the female range. When she comes in for her checkups, what parameters should be measured?

*Estradiol, testosterone, potassium (spironolactone side effect), BP (spironolactone side effect), blood glucose, prolactin, triglycerides*

At the conclusion of her exam, she mentions that she would like some feminization surgeries in the future. You counsel her on various options including facial feminization surgeries, breast augmentation, and genital reconstruction surgeries like vaginoplasty and orchiectomy (removal of the testes). How do surgeries affect screening practices? Consider both transmen and transwomen.

*Screening for adult natal male (typically after age 50): bone density, prostate cancer, testicular cancer, colorectal cancer*

*Screening for adult natal female (typically after age 50): bone density, breast cancer, cervical cancer, colorectal cancer*

*Key Points*

- *Check for the organs that a person has REGARDLESS of hormone therapy*
- *There are some recommendations that transwomen should get breast cancer screening due to the exposure of estrogen*

**Video Case Presentation Key Points (Appendix J)**

This is meant to be a large group discussion whereby students ask the facilitators questions about the encounter they just watched. Other students are also encouraged to share their thoughts particularly regarding positives and negatives of the encounter.

Points to Highlight in Video

- Positives
  - Provider asked how patient would like to be addressed
  - Maintained sympathetic tone even when patient revealed vaginal bleeding
  - Asked specifically about depression and anxiety: important as LGBTQ+ populations have higher rates of mental illness
  - Ascertained whether patient was on gender-affirming hormone therapy and gauged patient understanding of why hormone therapy was started
  - Set clear expectations about patient comfort and what the plan of the visit is going to be
    - Reiterated that patient is encouraged to stop and ask questions at any point and that this is collaborative environment
    - If you have treated trans patients before, it can be helpful to mention that to a trans patient
  - Asked about social history: living situation, work, substance use, feeling safe at home
  - Gave patient early notice that provider would like to do a physical exam during the next appointment and allowed friend to come with patient
- Negatives (ask students if there are aspects they would change about the encounter)
  - Provider did not share own pronouns and ask patient for theirs
  - Provider directly asked patient if they have vaginal bleeding
  - Said patient had “period,” however apologized quickly after
    - Emphasize to students that even with years of clinical experience and training, mistakes may happen
  - Quickly jumped into asking patient about sex assigned at birth
  - Used some technical terms such as “ovulate”
  - Did not ask how patient was getting testosterone: due to social barriers in accessing care (e.g., finding a provider, cost, lack of insurance), patients may get testosterone from other sources

Facilitator-prepared Questions

*Below are some facilitator-prepared questions to ensure similar topics are covered compared to the written case discussion. Students may ask these questions of their own accord*

- How do you elicit a patient’s understanding of their hormone therapy?
- What parts of the past medical history is key when interviewing a trans patient?
- Why did you specifically ask about mental health?
- Why did you focus on the social history?
- How would you provide age-appropriate health screening for this patient?
- How would you monitor the side effects of someone on hormone therapy?

*These are other questions that we had prepared to create more discussion about the provider’s thought process through the encounter. Students may ask these questions of their own accord*

- Some patients might not be comfortable sharing things even if they are essential to your differential. How do you navigate that?
- Why did you choose to ask about sex assigned at birth in the time that you did?
- How do you address making mistakes in the interview?
- How do you determine what is “essential” to treating and what is “useful but not essential” without making assumptions about the patient?

*If trans individuals are available to share their perspectives, the following questions can be asked*

- In your experience, what have been things that clinicians have done that made you feel more comfortable? Less comfortable?
- How can the clinician make a good first impression?
- What are changes you would have made to this encounter?
