# Supplementary material for: Teaching Trans-Centric Curricular Content Using Modified Jigsaw
Source: MedEdPORTAL. 2022 May 24;18:11257. doi: 10.15766/mep_2374-8265.11257 (PMC9127030; doi:10.15766/mep_2374-8265.11257)
Supplement: Supplementary file 1 — Activity and Materials Outline.docxFacilitator Guide.docxPresession Survey.docxPretest Questions.docxStudent Packet 1.docxStudent Packet 2.docxStudent Packet 3.docxStudent Packet 4.docxStudent Packet 5.docxSimulated Transgender Patient Interview.mp4Posttest Questions.docxPosttest Answers.docxPostsession Survey.docx [file mep_2374-8265.11257-s001.zip › C. Presession Survey.docx]

Thank you for taking this survey! Before we begin, we want to remind you NOT to include any personally identifying information in any of your responses. We also want to remind you that all responses will remain anonymous, so please answer honestly. We’ll begin by asking some questions about your prior experience.

1. How knowledgeable are you about the transgender community?

1 – Not at all knowledgeable

2 – Slightly knowledgeable

3 – Somewhat knowledgeable

4 – Moderately knowledgeable

5 – Extremely knowledgeable

1. Do you have personal experience interacting with transgender individuals?

Yes

No

1. Could you describe the context in which you knew the individual(s)? Did you note any societal obstacles they had to face?
2. Do you have personal experience interacting with transgender individuals **specifically in a healthcare setting?**

Yes

No

1. Could you describe the experience? Did you note any specific obstacles in their care?

Now we’ll ask about your knowledge of different topics related to transgender healthcare. Use the following scale (0-100) to rate your degree of confidence^1^:

0 10 20 30 40 50 60 70 80 90 100

Cannot Moderately Highly certain

do at all can do can do

1. Discussing gender identity with a peer?
2. Discussing gender identity with a patient?
3. Discussing gender affirming hormone therapy with a peer?
4. Discussing gender affirming hormone therapy with a patient?
5. Discussing clinical care for transgender patients with a peer?
6. Discussing clinical care for transgender patients with a patient?
7. Eliciting patient histories from transgender patients?
8. What are 3 things that should be asked when starting the interview with transgender patients?

Next we’ll ask about your opinion of clinical healthcare practices relating to transgender patients.

1. Transgender patients deserve the same level of quality care from medical institutions as cisgender patients.^2^

1 – Strongly disagree

2 – Disagree

3 – Disagree somewhat

4 – Neither agree nor disagree

5 – Agree somewhat

6 – Agree

7 – Strongly agree

1. Transgender patients should only seek health care from transgender health clinics.^2^

1 – Strongly disagree

2 – Disagree

3 – Disagree somewhat

4 – Neither agree nor disagree

5 – Agree somewhat

6 – Agree

7 – Strongly agree

1. All physicians have a responsibility to treat transgender patients.^2^

1 – Strongly disagree

2 – Disagree

3 – Disagree somewhat

4 – Neither agree nor disagree

5 – Agree somewhat

6 – Agree

7 – Strongly agree

1. Would you be comfortable if you became known among your professional peers as a doctor that cares for transgender patients?^2^

1 – Not at all comfortable

2 – Slightly comfortable

3 – Somewhat comfortable

4 – Moderately comfortable

5 – Extremely comfortable

1. Would you be comfortable if other patients that treat learned that you were treating transgender patients?^2^

1 – Not at all comfortable

2 – Slightly comfortable

3 – Somewhat comfortable

4 – Moderately comfortable

5 – Extremely comfortable

Lastly, we’ll ask about transgender medicine in medical education.

1. I am interested in learning about caring for transgender patients.

1 – Strongly disagree

2 – Disagree

3 – Somewhat disagree

4 – Neither agree nor disagree

5 – Somewhat agree

6 – Agree

7 – Strongly agree

1. Content about transgender populations should be incorporated into medical school education.

1 – Strongly disagree

2 – Disagree

3 – Somewhat disagree

4 – Neither agree nor disagree

5 – Somewhat agree

6 – Agree

7 – Strongly agree

1. Learning about gender affirming hormone therapy will help you gain a better understanding of Endocrine/Reproduction concepts.

1 – Strongly disagree

2 – Disagree

3 – Disagree somewhat

4 – Neither agree nor disagree

5 – Agree somewhat

6 – Agree

7 – Strongly agree

Thank you for your participation!

^1^Adapted from Bandura’s 100-point self-efficacy scale: Bandura A. Guide for Constructing Self-Efficacy Scales. In: Pajares J, Urdan T. *Self-Efficacy Beliefs of Adolescents*. United States: Information Age Publishing, Inc; 2006:307-337.

^2^Adapted from Sanchez, et al. (2006): Sanchez NF, Rabatin J, Sanchez JP, Hubbard S, Kalet A. Medical students' ability to care for lesbian, gay, bisexual, and transgendered patients. *Fam Med*.2006;38(1):21-27.
