# Supplementary material for: Teaching Trans-Centric Curricular Content Using Modified Jigsaw
Source: MedEdPORTAL. 2022 May 24;18:11257. doi: 10.15766/mep_2374-8265.11257 (PMC9127030; doi:10.15766/mep_2374-8265.11257)
Supplement: Supplementary file 1 — Activity and Materials Outline.docxFacilitator Guide.docxPresession Survey.docxPretest Questions.docxStudent Packet 1.docxStudent Packet 2.docxStudent Packet 3.docxStudent Packet 4.docxStudent Packet 5.docxSimulated Transgender Patient Interview.mp4Posttest Questions.docxPosttest Answers.docxPostsession Survey.docx [file mep_2374-8265.11257-s001.zip › M. Postsession Survey.docx]

Thank you for participating in the transgender and gender affirming hormone therapy session! Before we begin, we want to remind you NOT to include any personally identifying information in any of your responses. We want to gauge the impact the session has on you. We want to remind you that all responses will remain anonymous, so please answer honestly. We’ll begin by asking some questions about your knowledge of different topics related to transgender healthcare.

Use the following scale (0-100) to rate your degree of confidence:

0 10 20 30 40 50 60 70 80 90 100

Cannot Moderately Highly certain

do at all can do can do

1. Discussing gender identity with a peer?
2. Discussing gender identity with a patient?
3. Discussing gender affirming hormone therapy with a peer?
4. Discussing gender affirming hormone therapy with a patient?
5. Discussing clinical care for transgender patients with a peer?
6. Eliciting patient histories from transgender patients?
7. What are 3 things that should be asked when starting the interview with transgender patients?

Next we’ll ask you a few questions about your opinion of clinical healthcare practices relating to transgender patients.

1. Transgender patients deserve the same level of quality care from medical institutions as cisgender patients.

1 – Strongly disagree

2 – Disagree

3 – Disagree somewhat

4 – Neither agree nor disagree

5 – Agree somewhat

6 – Agree

7 – Strongly agree

1. Transgender patients should only seek health care from transgender health clinics.

1 – Strongly disagree

2 – Disagree

3 – Disagree somewhat

4 – Neither agree nor disagree

5 – Agree somewhat

6 – Agree

7 – Strongly agree

1. All physicians have a responsibility to treat transgender patients.

1 – Strongly disagree

2 – Disagree

3 – Disagree somewhat

4 – Neither agree nor disagree

5 – Agree somewhat

6 – Agree

7 – Strongly agree

1. Would you be comfortable if you became known among your professional peers as a doctor that cares for transgender patients?

1 – Not at all comfortable

2 – Slightly comfortable

3 – Somewhat comfortable

4 – Moderately comfortable

5 – Extremely comfortable

1. Would you be comfortable if other patients that treat learned that you were treating transgender patients?

1 – Not at all comfortable

2 – Slightly comfortable

3 – Somewhat comfortable

4 – Moderately comfortable

5 – Extremely comfortable

Lastly, we’ll ask you a few specific questions about the session itself and transgender medicine in medical education.

1. I am interested in learning more about caring for transgender patients.

1 – Strongly disagree

2 – Disagree

3 – Somewhat disagree

4 – Neither agree nor disagree

5 – Somewhat agree

6 – Agree

7 – Strongly agree

1. Content about transgender populations should be incorporated into medical school education.

1 – Strongly disagree

2 – Disagree

3 – Somewhat disagree

4 – Neither agree nor disagree

5 – Somewhat agree

6 – Agree

7 – Strongly agree

1. This session helped me gain a better understanding of Endocrine/Reproduction concepts.

1 – Strongly disagree

2 – Disagree

3 – Somewhat disagree

4 – Neither agree nor disagree

5 – Somewhat agree

6 – Agree

7 – Strongly agree

1. This session helped me gain a better understanding of caring for transgender patients.

1 – Strongly disagree

2 – Disagree

3 – Somewhat disagree

4 – Neither agree nor disagree

5 – Somewhat agree

6 – Agree

7 – Strongly agree

1. This session will help me be a better clinician in the future.

1 – Strongly disagree

2 – Disagree

3 – Somewhat disagree

4 – Neither agree nor disagree

5 – Somewhat agree

6 – Agree

7 – Strongly agree

1. The peer-teaching style of the session to worked well.

1 – Strongly disagree

2 – Disagree

3 – Somewhat disagree

4 – Neither agree nor disagree

5 – Somewhat agree

6 – Agree

7 – Strongly agree

1. There was an adequate amount of time allotted for the material.

1 – Strongly disagree

2 – Disagree

3 – Somewhat disagree

4 – Neither agree nor disagree

5 – Somewhat agree

6 – Agree

7 – Strongly agree

1. I thought the difficulty of the material was appropriate.

1 – Strongly disagree

2 – Disagree

3 – Somewhat disagree

4 – Neither agree nor disagree

5 – Somewhat agree

6 – Agree

7 – Strongly agree

1. I think this session should be incorporated into the formal curriculum for first-year students.

1 – Strongly disagree

2 – Disagree

3 – Somewhat disagree

4 – Neither agree nor disagree

5 – Somewhat agree

6 – Agree

7 – Strongly agree

1. Do you have any other feedback that would help us improve the session?

Thank you for participating in this session!
